# Palmitoyl-carnitine Regulates Lung Development by Promoting Pulmonary Mesenchyme Proliferation

**DOI:** 10.34133/research.0620

**Published:** 2025-03-18

**Authors:** Xing Liu, Sin Man Lam, Yu Zheng, Lesong Mo, Muhan Li, Tianyi Sun, Xiaohui Long, Shulin Peng, Xinwei Zhang, Mei Mei, Guanghou Shui, Shilai Bao

**Affiliations:** ^1^ State Key Laboratory of Molecular Developmental Biology, Institute of Genetics and Developmental Biology, Chinese Academy of Sciences, Beijing 100101, China.; ^2^ School of Life Sciences, University of Chinese Academy of Sciences, Beijing 100049, China.; ^3^ Department of Respiratory, Children’s Hospital of Nanjing Medical University, Nanjing, China.; ^4^ Guangzhou National Laboratory, Guangzhou, Guangdong 510005, China.; ^5^ Department of Hematology Oncology Center, Beijing Children’s Hospital, Capital Medical University, Beijing, China.

## Abstract

Disruption of acylcarnitine homeostasis results in life-threatening outcomes in humans. Carnitine–acylcarnitine translocase deficiency (CACTD) is a scarce autosomal recessive genetic disease and may result in patients’ death due to heart arrest or respiratory insufficiency. However, the reasons and mechanism of CACTD inducing respiratory insufficiency have never been elucidated. Herein, we employed lipidomic techniques to create comprehensive lipidomic maps of entire lungs throughout both prenatal and postnatal developmental stages in mice. We found that the acylcarnitines manifested notable variations and coordinated the expression levels of carnitine–acylcarnitine translocase (Cact) across these lung developmental stages. *Cact*-null mice were all dead with a symptom of respiratory distress and exhibited failed lung development. Loss of Cact resulted in an accumulation of palmitoyl-carnitine (C16-acylcarnitine) in the lungs and promoted the proliferation of mesenchymal progenitor cells. Mesenchymal cells with elevated C16-acylcarnitine levels displayed minimal changes in energy metabolism but, upon investigation, revealed an interaction with sterile alpha motif domain and histidine-aspartate domain-containing protein 1 (Samhd1), leading to decreased protein abundance and enhanced cell proliferation. Thus, our findings present a mechanism addressing respiratory distress in CACTD, offering a valuable reference point for both the elucidation of pathogenesis and the exploration of treatment strategies for neonatal respiratory distress.

## Introduction

As an essential part of the mammalian respiratory system, the fundamental task of the lung is to facilitate the interchange of gases between the organism and the external environment [1–4]. Lung organogenesis commences at embryonic day 9.5 (E9.5) and extends through postnatal day 30 (P30), ultimately culminating in the intricate structuring and functional proficiency of pulmonary tissue [5–8]. Based on alterations in airway morphology, the stages of lung development have traditionally been divided into 4 main phases: pseudoglandular, canalicular, saccular, and alveolar [7]. During this process, the lung epithelium separates into 2 distinct components: the distal airways, which give rise to alveoli for gas exchange, and the proximal airways, which develop into the bronchi and bronchioles. By E16.5, the distal airways begin to differentiate, resulting in the formation of pre-alveolar structures referred to as canaliculi and saccules, consisting of alveolar type II (ATII) cells that produce surfactant and alveolar type I (ATI) cells that are specialized for gas exchange [9]. These structures facilitate gas exchange in neonates until complete alveolarization takes place postnatally [9]. In addition to epithelia, platelet-derived growth factor receptor α-expressing (Pdgfrα^+^) cells have been identified as mesenchymal progenitors in the pulmonary environment [10]. In the pseudoglandular stage, distinct populations of Pdgfrα^+^ cells were observed surrounding the proximal airways [11]. Pdgfrα^+^ cells emerged within the more distal mesenchyme in the canalicular stage [11]. Previous research more precisely characterized the Pdgfrα^+^ cell lineage, revealing their propensity to differentiate into both lipofibroblasts and myofibroblasts (MYFs) during the advanced phases of lung development [11]. These findings substantiate the significance of Pdgfrα^+^ cells in the development and maintenance, as well as the pathogenesis, of the mesenchymal framework [12–17]. Our findings, supported by studies from others, have established that lung mesenchymal progenitors are pivotal regulators of lung development and key contributors to the progression of pulmonary diseases [16,18].

Substantial evidence demonstrates that lipid metabolism intricately regulates the development and regeneration of various organs [19–21]. Lipid metabolism regulation fundamentally underpins the developmental processes of the brain, heart, and liver [22–25]. Additionally, lipids decisively dictate the fate of embryonic stem cells, T helper cell lineages, skeletal progenitor cell lineages, and fibroblasts [26–30]. The lung is infrequently recognized as a vital organ for lipid metabolism. However, lipid metabolism does participate in pulmonary disease. As is well-known, phospholipids serve as a primary component of pulmonary surfactant, efficiently lowering surface tension at the air/liquid interface in alveoli and averting lung collapse [31]. Imbalance in surfactant homeostasis can contribute to chronic obstructive pulmonary disease, idiopathic pulmonary fibrosis, acute respiratory distress syndrome (RDS), infections, and a range of other respiratory ailments [32–35].

Fatty acid oxidation (FAO), a crucial process in lipid metabolism, provides energy, regulates lipid homeostasis, supports mitochondrial function, and contributes to metabolic flexibility, comprising pivotal enzymes such as carnitine palmitoyl transferase 1 (Cpt1), carnitine–acylcarnitine translocase (Cact), and carnitine palmitoyl transferase 2 (Cpt2). Cpt1 deficiency promotes cardiac regeneration in adult mice by fostering the accumulation of α-ketoglutarate within cardiomyocytes, thereby triggering the activation of α-ketoglutarate-dependent lysine demethylase KDM5 [36,37]. Cpt2 deficiency potentiates endothelial-to-mesenchymal transition through regulation of intracellular acetyl-coenzyme A (CoA) levels and suppressor of mothers against decapentaplegic homolog 7 (SMAD7) signaling [22]. It has been documented that the majority of patients afflicted with carnitine–acylcarnitine translocase deficiency (CACTD) endure severe RDS during infancy, characterized by a rapid disease progression and notably high mortality rates [38–41]. CACTD patients exhibit mitochondrial dysfunction during long-chain FAO, resulting in the accumulation of long-chain acylcarnitines, especially C16-acylcarnitine [42]. In clinical settings, the elevated C16-acylcarnitine has been shown to not only activate the skeletal muscle sarcoplasmic reticulum Ca^2+^ release channel but also induce endothelial cells to release prostacyclin, thereby altering vascular endothelial function [43–47]. However, the mechanisms of Cact and C16-acylcarnitine that underpin the spatiotemporal regulation of developmental signaling cues throughout the progression of symptoms in CACTD patients remain unclear.

Here, we performed an extensive analysis of the lipidomic profile and dynamic modifications throughout the course of lung development. It is worth noting that during the pseudoglandular stage, there is a marked elevation in acylcarnitine levels, which subsequently decline notably during the canalicular stage, implicating acylcarnitine in lung development. Due to the consistent correlation between the expression pattern of Cact and the fluctuation pattern in acylcarnitine levels, our utilization of mice with loss of *Cact* function revealed the accumulation of C16-acylcarnitine, resulting in lung defects and subsequent symptoms of respiratory distress. Our findings demonstrate that the accumulated C16-acylcarnitine binds to sterile alpha motif domain and histidine-aspartate domain-containing protein 1 (Samhd1) in *Cact*-deficient mice, stimulating the proliferation of mesenchymal progenitors instead of causing energy disruption. Our study provides a new target for therapy of neonatal RDS.

## Results

### Lung lipidome analysis and coverage

**Fig. 1.**
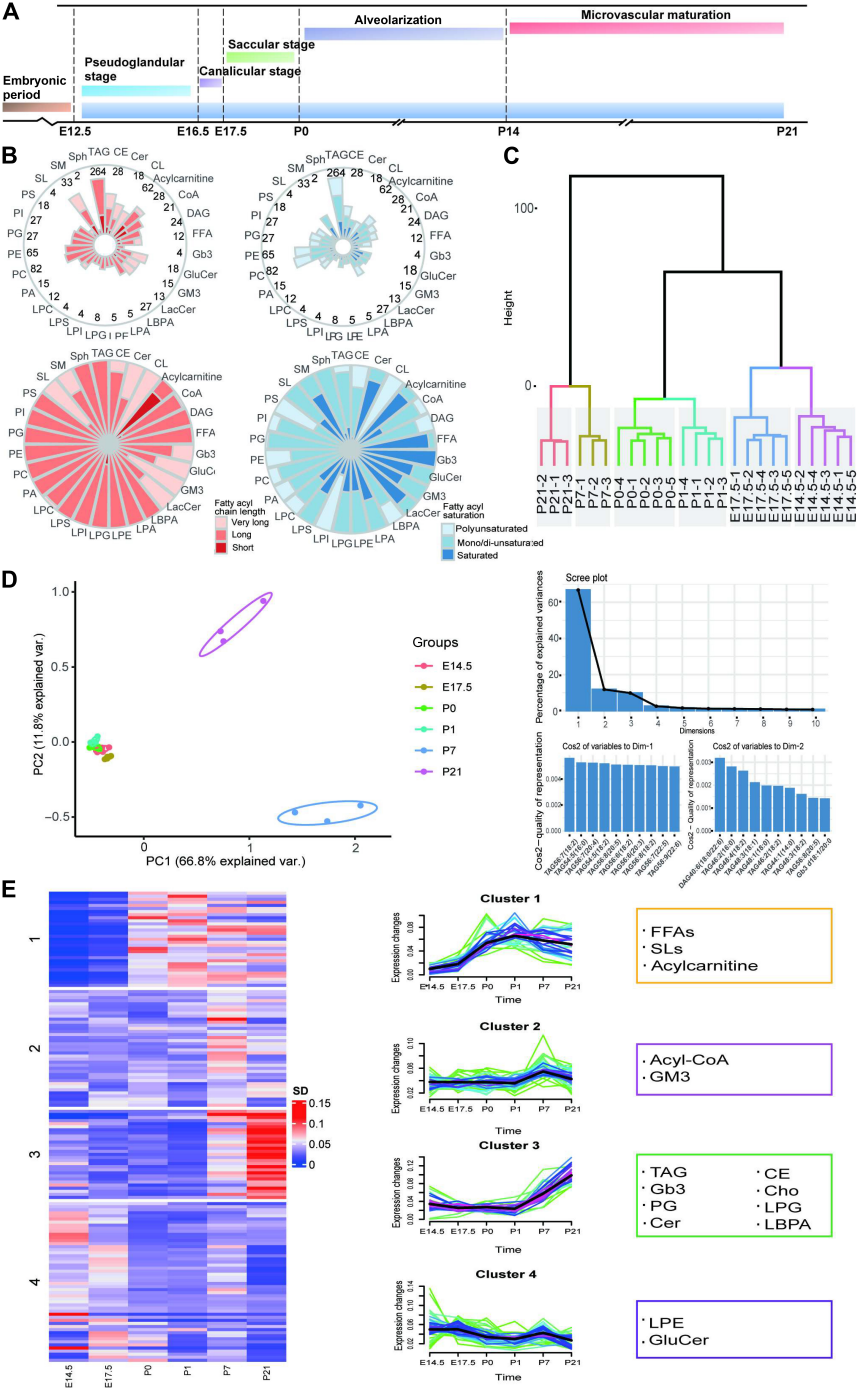
A quantitative analysis of homogenized liposome profiles during mouse lung development was conducted using mass spectrometry. (A) Whole lungs were collected at 6 time points of prenatal and postnatal lung development (including E14.5, E17.5, P0, P1, P7, and P21). *n* = 4. E, embryonic; P, postnatal. (B) In the lipidome of the lung tissue under investigation, a total of 845 lipids, categorized into 28 distinct lipid classes, were identified and precisely quantified. To visually depict the distribution of fatty acyl groups, characterized by varying carbon chain lengths and degrees of unsaturation, across the primary lipid classes in the E14.5 lung lipidome, radar plots were utilized. The numerical values displayed along the circumferential axes of the radar plot in the upper panel signify the abundance of quantitative lipid species within each respective lipid class. (C) The dendrogram analysis of individual samples unveiled substantial intergroup disparities, accompanied by minimal intragroup variations. (D) The principal component analysis (PCA) results for a solitary sample are presented, with the figures in parentheses indicating the proportion of the total variance accounted for by each principal component. The adjacent bar charts delineate the 10 variables that exhibit the greatest contribution to each component (namely, dimension-1 and dimension-2), respectively. (E) A hierarchical clustering algorithm was utilized to classify lipid species into 4 distinct clusters, based on their observed compositional changes during lung development. The left panel portrays the row-normalized abundance (repeat median) of lipid species that exhibit significant temporal fluctuations throughout lung ontogeny. The middle panel illustrates the variations in individual lipid species across different samples, arranged in accordance with specified developmental stages. The right panel highlights the representative lipids within each cluster, delineated and enclosed within boxed areas for clarity. FFAs, free fatty acids; SLs, sulfatides; CoA, coenzyme A; GM3, monosialodihexosylganglioside; TAG, triacylglycerol; Gb3, globotriaosylceramide; PG, phosphatidylglycerol; Cer, ceramide; CE, cholesteryl ester; Cho, cholesterol; LPG, lysophosphatidylglycerol; LBPA, lysobisphosphatidic acid; LPE, lysophosphatidylethanolamine; GluCer, glucosylceramide.

To unravel the composition and dynamics of lipids throughout mouse lung development, we conducted a comprehensive analysis of lung lipidomics at pivotal developmental stages E14.5, E17.5, P0, P1, P7, P14, and P21, utilizing a liquid chromatography–tandem mass spectrometry (LC–MS/MS) approach (Fig. 1A). We detected 845 lipids distributed across 28 lipid subclasses, characterized by diverse fatty acyl chain lengths and degrees of saturation (Fig. 1B to D and Figs. S1 to S3). We observed elevated levels of acylcarnitine, free fatty acids (FFAs), and sulfatides (SLs) during the pseudoglandular stage, with a subsequent decline in other stages (Fig. 1E and Fig. S1A). The concentrations of acyl-CoA and monosialodihexosylganglioside (GM3) were heightened in the canalicular and saccular stages and diminished in other stages (Fig. 1E and Fig. S1B). Triacylglycerols (TAGs), globotriaosylceramide (Gb3), phosphatidylglycerol (PG), ceramide, cholesteryl ester, cholesterol, lysophosphatidylglycerol (LPG), and lysobisphosphatidic acid exhibited a peak in the alveolar stage, contrasting with lower levels in other periods (Fig. 1E and Fig. S1C). In addition, lysophosphatidylethanolamine (LPE) and glucosylceramide (GluCer) demonstrated no significant variations across each stage of lung development (Fig. 1E and Fig. S1D). Taken together, our results elucidate a broad spectrum of alterations within the lipidomic landscape of the mouse lung, encompassing the stages from branching morphogenesis to alveolar development, providing a comprehensive and intricate overview of the temporal dynamics exhibited by various lipid species during lung development.

### The role of acylcarnitine in mouse lung explants

**Fig. 2.**
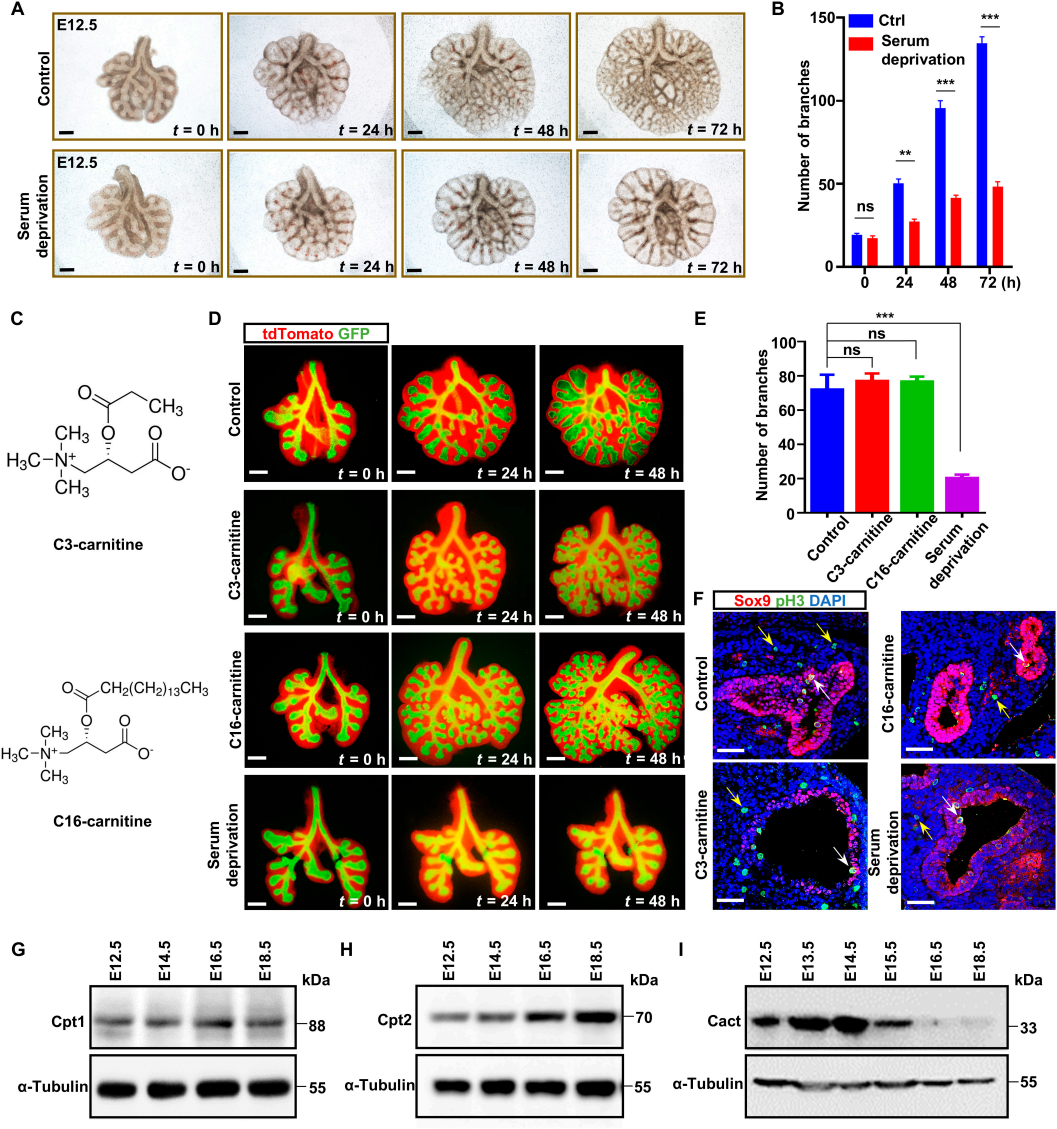
Acylcarnitine scarcity induces lung explants defects. (A) Whole-mount images of E12.5 explant lungs under normal conditions cultured or serum deprivation cultured immediately after dissection and after 0, 24, 48, and 72 h in culture. *n* = 6 per group. Scale bars: 200 μm. (B) Quantitative assessment of the branch count for the lung explants depicted in panel (A). (C) The structures of C3-carnitine and C16-carnitine. (D) Under serum-deprived culture conditions, C3-carnitine and C16-carnitine rescued the branching of the lung explant. The red signal represents mesenchymal cells; the green signal represents epithelial cells. Scale bars: 200 μm. (E) Quantitative analysis of the branch count for the lung explants illustrated in panel (D). (F) High-resolution confocal microscopic images depicting the immunostaining of tissue sections for the transcription factor SRY-box transcription factor 9 (Sox9) and the mitotic marker phosphorylated histone H3 (pH3) are presented. These images offer a detailed visualization of the spatial distribution and abundance of both proteins within the tissue sections. The red signal represents Sox9; the green signal represents pH3. Yellow arrowheads point to pH3^+^ mesenchymal cells; white arrowheads point to pH3^+^ cells in Sox9^+^ cells. Scale bars: 50 μm. (G to I) Carnitine palmitoyl transferase 1 (Cpt1), carnitine palmitoyl transferase 2 (Cpt2), and carnitine–acylcarnitine translocase (Cact) expression patterns were analyzed by western blot. Six biological replicates were obtained from 3 independent experiments. The data are presented as mean ± standard error of the mean (SEM). Statistical significance was determined using analysis of variance (ANOVA), with asterisks denoting the level of significance: *P < 0.05; **P < 0.01; ***P < 0.001. “ns” indicates that the difference was not statistically significant. GFP, green fluorescent protein; DAPI, 4′,6-diamidino-2-phenylindole.

Following a thorough analysis of the lipidomic data, it becomes apparent that the fluctuations in acylcarnitine levels during lung development are of utmost importance (Fig. 1E and Fig. S1A). Based on previous research, acylcarnitine emerges as an essential metabolite in FAO, assuming critical function in various cellular energy metabolism pathways [42]. To investigate further the significance of acylcarnitines in the development of the lungs, we harvested early fetal lungs for cultivation [48]. Branching morphogenesis was impaired, with a complete absence of new branch formation in the lungs subjected to serum deprivation, whereas normal branching patterns were observed in the control lungs (Fig. 2A and B). Given that serum contains a complex mixture of bioactive components, including lipids, amino acids, vitamins, and growth factors, the stunted branching observed under serum-deprived conditions may result from the combined effects of multiple deficiencies. However, as lipids are a major component of serum, their depletion might play a significant role in this phenotype. We observed that the addition of short-chain acylcarnitine (C3-acylcarnitine) and long-chain acylcarnitine (C16-acylcarnitine) to serum-deprived cultures restored normal branch morphogenesis by reinstating the normal proliferation patterns of epithelial and mesenchymal cells in the fetal lung (Fig. 2C to F).

Acylcarnitine intricately participates in mitochondrial FAO, a vital pathway for energy production [42]. Notably, our study demonstrates a notable correlation between the expression profiles of Cact and the variations in acylcarnitine levels throughout lung development (Fig. 2G to I). This indicates a crucial role for Cact in orchestrating the dynamics of acylcarnitine metabolism, thereby contributing significantly to the complex processes governing lung organogenesis and functional maintenance.

### Loss of Cact disrupts lung development in mice

**Fig. 3.**
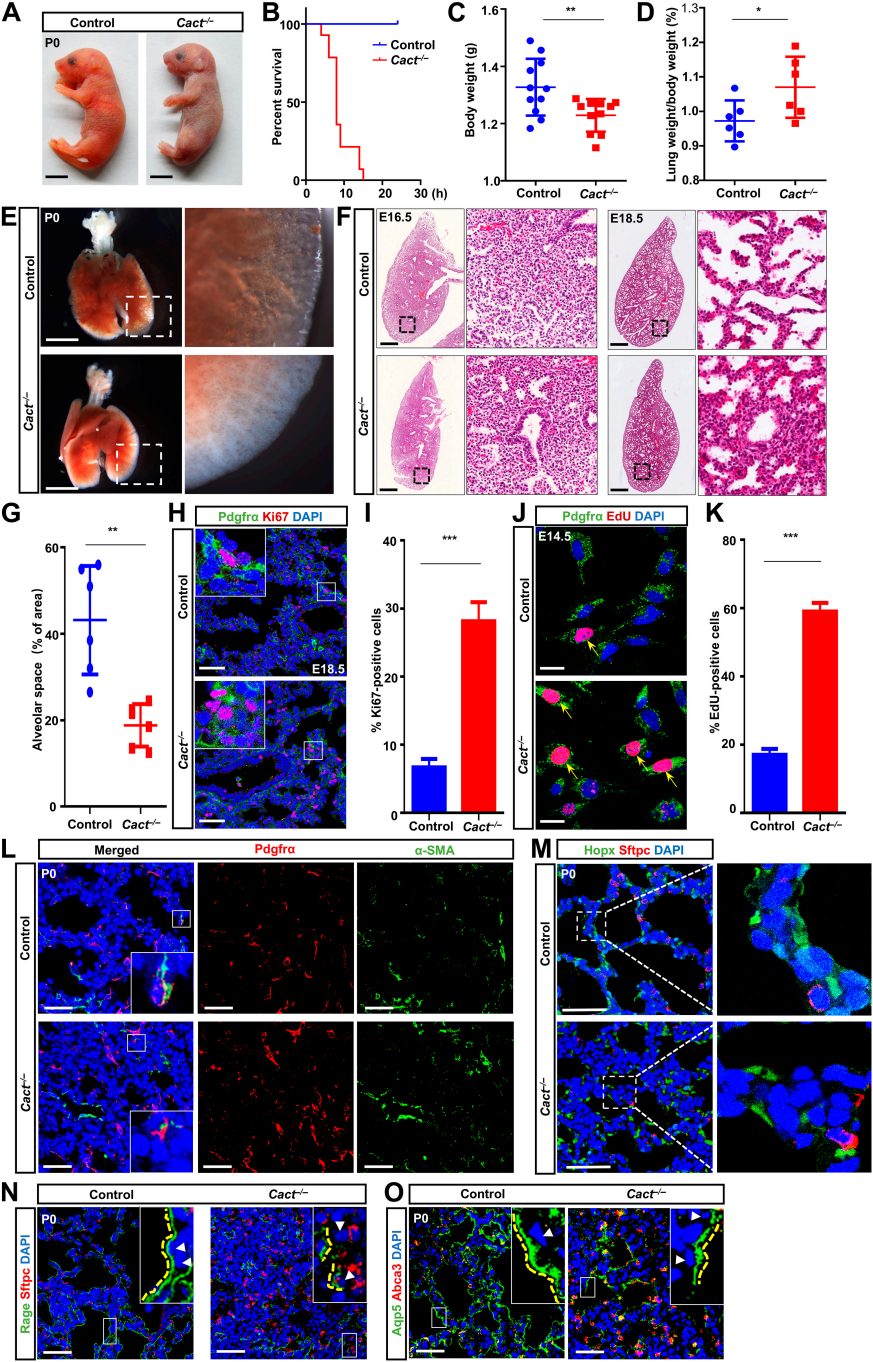
Inactivation of Cact exhibits lung development defects. (A) The intact pups of the control group and the Cact deletion group at the time of birth. Scale bars: 0.2 cm. (B) Life assay of control and *Cact^−/−^* mice. Mouse death is defined by the following criteria: the complete cessation of respiration, with no chest movements or respiratory effort, and the absence of a detectable heartbeat. Reflex responses are entirely lost, including the lack of withdrawal or escape reactions upon gentle stimulation of the tail or paw. Furthermore, the skin exhibits pallor or cyanosis, and the body temperature falls below 27 °C, confirming the loss of vital functions. A total of 11 samples (*n* = 11) were included in the quantification. (C) The body weights of the P0 control mice and the *Cact^−/−^* mice were measured and quantified. A total of 11 samples (*n* = 11) were included in the quantification. (D) The lung-to-body weight ratios of P0 control and *Cact^−/−^* mice were quantified. *n* = 6 per genotype used for quantification. (E) A ventral perspective of dissected lungs harvested from P0 control mice and *Cact*-deficient (*Cact^−/−^*) mice is depicted. The areas indicated by boxes in the main images are provided in magnified form as insets for detailed examination. Scale bars: 2.5 mm. (F) Hematoxylin and eosin (H&E) staining was performed on the lungs of control and *Cact^−/−^* mice at E16.5 and E18.5. The boxed regions are enlarged in the insets. Scale bars: 500 μm. (G) Morphometric analysis of alveolar space in control and *Cact^−/−^* E18.5 lungs. The experiment represent *n* = 6 biological replicates. (H) Immunofluorescence staining for Ki67 and platelet-derived growth factor receptor α (Pdgfrα) was conducted on lung tissues from control and *Cact^−/−^* mice at E18.5. The boxed areas are shown at higher magnification in the insets. Scale bars: 50 μm. (I) Quantitative analysis of proliferation shown in panel (H) as shown. *n* = 5 mice per group. (J) 5-Ethynyl-2′-deoxyuridine (EdU) assay of Pdgfrα^+^ cells. Arrows point to EdU and Pdgfrα double-positive cells. Scale bars: 20 μm. (K) Quantitative analysis of the percentage of EdU-positive (EdU^+^) cells presented in panel (J) Quantification was carried out in *n* = 5 samples. (L) Immunostaining for Pdgfrα and alpha smooth muscle actin (α-SMA) on control (*n* = 10) and *Cact^−/−^* (*n* = 10) P0 lung sections. The regions outlined by boxes are provided in magnified detail in the insets. Scale bars: 50 μm. (M) Immunostain of lung sections from control or *Cact^−/−^* mice with HOP homeobox (Hopx) and surfactant protein C (Sftpc). The image displayed on the right is a magnified representation of the region indicated by the dashed white box. Scale bars: 50 μm. (N) Immunohistochemical staining for receptor for advanced glycation endproducts (Rage) and Sftpc was performed on lung sections from P0 mice. Scale bars: 50 μm. (O) Immunostaining for aquaporin 5 (Aqp5) and adenosine triphosphate (ATP)-binding cassette subfamily A member 3 (Abca3) was conducted on lung sections from both control (*n* = 8 per stage) and Cact-knockout (*Cact^−/−^*) mice (*n* = 8 per stage) at postnatal day 0 (P0). Panels (N) and (O) display magnified views of the regions delineated by the white boxes. Arrowheads indicate the nuclei of alveolar type I (ATI) cells, while yellow dotted lines delineate the morphology of ATI cells. Scale bars are set at 50 μm. Data are presented as mean ± SEM. Statistical significance is denoted as *P < 0.05, **P < 0.01, and ***P < 0.001, based on the Student *t* test.

To address the underlying mechanism of CACTD, we generated a *Cact* knockout mice (*Cact^−/−^*) (Fig. 3A). Remarkably, our results indicate that a deficiency in Cact does not elicit any discernible anomalies in vital organs, including the brain, liver, and heart (Fig. S4A and B). Compared to their littermates, *Cact^−/−^* neonates presented with respiratory distress accompanied by a cyanotic phenotype (Fig. 3A). Notably, during natural delivery, no *Cact^−/−^* pups survived beyond 24 h (Fig. 3B). These pups exhibited complete respiratory arrest, characterized by the absence of chest movements or respiratory effort, a lack of detectable heartbeat, and the loss of all reflex responses. A notable reduction in body weight was also recorded (Fig. 3C and D). A preliminary macroscopic assessment of P0 *Cact^−/−^* fetal lungs revealed a marked reduction in lobular size, accompanied by occasional instances of fusion, while the overall lobular architecture and quantity remained intact (Fig. 3E). The genotype distribution of all offspring follows Mendelian inheritance principles, suggesting the absence of embryonic lethality (Table). Subsequent histological and morphological analyses indicated a reduction in alveolar space and a concomitant increase in the thickness of adjacent alveolar septa in *Cact^−/−^* mice compared to that in controls (Fig. 3F and G).

**Table.** The proportion of offspring genotypes from *Cact^+/−^* and *Cact^+/−^* mice mating at different periods

Age	No. of litters	Total mice	*Cact^−/−^*	*Cact^+/−^*	*Cact^+/+^*
E14.5	5	64	21 (31.3%)	31 (48.4%)	15 (23.4%)
E18.5	5	57	11 (19.3%)	28 (49.1%)	16 (28.1%)
P0	5	54	13 (24.1%)	32 (59.2%)	9 (16.7%)

To further elucidate the etiology behind the documented enhancement in tissue compactness in *Cact^−/−^* lungs, we systematically explored 3 potential contributing factors: escalated cell proliferation, modified apoptosis, or impaired differentiation. Our results showed no significant change in the quantity of apoptotic cells within the mutant lungs relative to that in the wild type, in either epithelial or mesenchymal cell populations (Fig. S4C and D). Subsequently, we conducted immunostaining utilizing the proliferative nuclear marker Ki67 to quantify positive cells at E18.5. Remarkably, mesenchymal cell proliferation exhibited a pronounced increase in mutant lungs compared to that in the wild type (Fig. 3H and I), a phenomenon not observed in epithelial cells (Fig. S4E and F). In isolated *Cact^−/−^* MYFs, a consistent and significant increase in proliferation was detected in *Cact^−/−^* cells (Fig. 3J and K). Immunofluorescence staining results depicting enhanced Ki67 expression in cultured *Cact^−/−^* cells mirrored the phenotype of *Cact^−/−^* mice (Fig. S4G and H). Following live cell labeling and counting, *Cact^−/−^* mesenchymal cells were found to be significantly increased compared to wild-type ones (Fig. S4I). These findings suggest that *Cact* is indispensable for the normal proliferation of the Pdgfrα^+^ progenitor.

To demonstrate the impact of Cact loss on cell differentiation during lung development, we conducted a detailed analysis of the differentiation status of mutant epithelial and mesenchymal cells by staining for representative marker proteins. At P0, mesenchymal progenitors (Pdgfrα^+^ cells) and various proximal airway epithelial cell types, including basal cells (Krt5^+^ [Krt5: keratin 5]), neuroendocrine cells (CGRP^+^ [CGRP: calcitonin gene-related peptide]), and ciliated cells (Ac-tubulin^+^), as well as proximal (Sox2^+^ [Sox2: SRY-box transcription factor 2]) and distal progenitors (Sox9^+^ [Sox9: SRY-box transcription factor 9]), were all detected in *Cact^−/−^* lungs (Fig. 3L and Fig. S5A to D). We observed that the ATI cells in the wild-type lung extensively covered the alveolar surface, exhibiting a planar or flattened-out appearance at both E18.5 and P0, whereas the majority of ATI cells in the *Cact^−/−^* lung were densely packed and unflattened (Fig. 3M to O). However, the differentiation of ATI and ATII cells was identical in both control and *Cact^−/−^* mice at E18.5 (Fig. 3M). Further, we found more collagen-producing mesenchymal stromal cells in Cact-deficient lung tissue, which leads to impaired ATI cell morphology (Fig. S5E to H). Consequently, these results suggest that the deficiency of Cact display abnormal proliferation of mesenchymal progenitors, accompanied by anomalous morphology of ATI cells, contributing to lung defects.

### Mesenchymal Cact ablation disrupts mouse lung development

To ascertain the precise cellular prerequisites for the role of Cact in mouse lung maturation, we utilized a floxed *Cact^fl/fl^* strain. Initially, we specifically abrogated *Cact* manifestation in the epithelial cells via the *Sonic hedgehog (Shh) Cre* line (hereafter *Shh;Cact*) and, intriguingly, observed no apparent respiratory dysfunction or lung defects (Fig. S6A to I) [49].

**Fig. 4.**
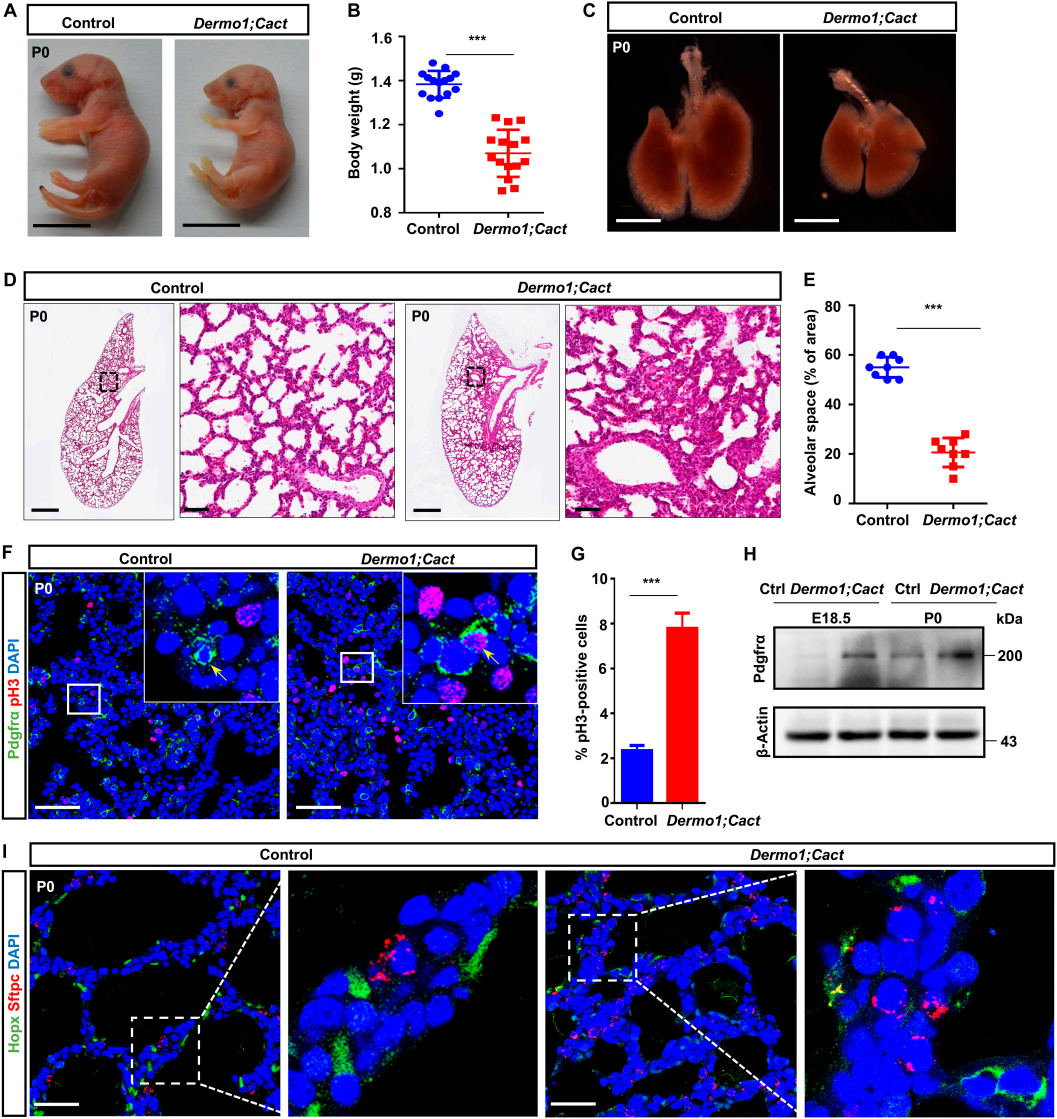
Mesenchyme-specific *Cact* deletion disrupts lung development. (A) Gross morphological analysis was conducted on P0 mice, including both control animals (*n* = 24) and those with the *Dermo1;Cact* genotype (*n* = 22). Scale bars: 1 cm. (B) The body weights of the P0 control and *Dermo1;Cact* mutant were quantified. *n* = 9 per genotype were used for this quantification. (C) A ventral view of the dissected lungs is presented for both P0 control mice and *Dermo1;Cact* mutant mice. Scale bars: 2.5 mm. (D) H&E staining of control and *Dermo1;Cact* lungs at P0. Boxed regions are magnified in insets. Scale bars: 500 μm. (E) A morphometric analysis was conducted on the alveolar space in control (*n* = 15) and *Dermo1;Cact* (*n* = 15) P0 lungs. (F) Immunofluorescence staining of pH3 and Pdgfrα in lung tissues at P0 from control and *Dermo1;Cact* mice. The areas enclosed within the boxes are displayed in magnified format in the insets. Scale bars: 50 μm. (G) Quantitative analysis of proliferation shown in panel (F) as shown. *n* = 5 mice per group. (H) Western blot analysis showing the boundary expressed Pdgfrα in control and *Dermo1;Cact* lung tissues. (I) Immunostain of lung sections from control or *Dermo1;Cact* mice with Hopx and Sftpc. The images displayed on the right are magnified views of the regions delineated by the dashed white boxes. Scale bars: 50 μm. The data are presented as mean ± SEM, with statistical significance denoted as ***P < 0.001, based on the Student *t* test.

To further assess *Cact* function in the interstitial tissue of lung, we bred *Dermo1-Cre* with *Cact^fl/fl^* mice (hereafter *Dermo1;Cact*) [50]. Notably, *Dermo1;Cact* mice exhibited a significant reduction in body size and weight compared to their littermates (Fig. 4A to C), mirroring the phenotype observed in *Cact^−/−^* mice. Additionally, *Dermo1;Cact* mice displayed a smaller size and alveolar collapse (Fig. 4D and E). Noteworthy, *Dermo1;Cact* mice exhibited an elevated count of proliferating mesenchymal cells (PH3^+^Pdgfrα^+^/Pdgfrα^+^) and displayed aberrant morphology in ATI cells (Fig. 4F to I). Essentially, conditionally knocking out *Cact* within the lung mesenchyme led to pulmonary abnormalities, mirroring observations seen in the conventional knockout of *Cact* mice. Consequently, these results emphasize the critical requirement of Cact in lung mesenchyme for maintaining normal proliferation of mesenchymal progenitors, essential for proper lung development and establishment of functional respiratory organs.

To further ascertain whether the absence of Cact expression is implicated in the aforementioned lung abnormalities, we introduced Cact supplementation into discrete MYF cultures. Consistently, MYFs derived from the *Cact*-deficient lung exhibited a significant increase in proliferation (Fig. S6J and K). Notably, restoring Cact expression to control levels effectively rescued the proliferation of mutant cells, providing solid evidence that the deficiency in Cact expression is indeed responsible for the observed proliferation defect in mice.

### Loss of Cact in the lungs induces an accumulation of elongated acylcarnitine chains

**Fig. 5.**
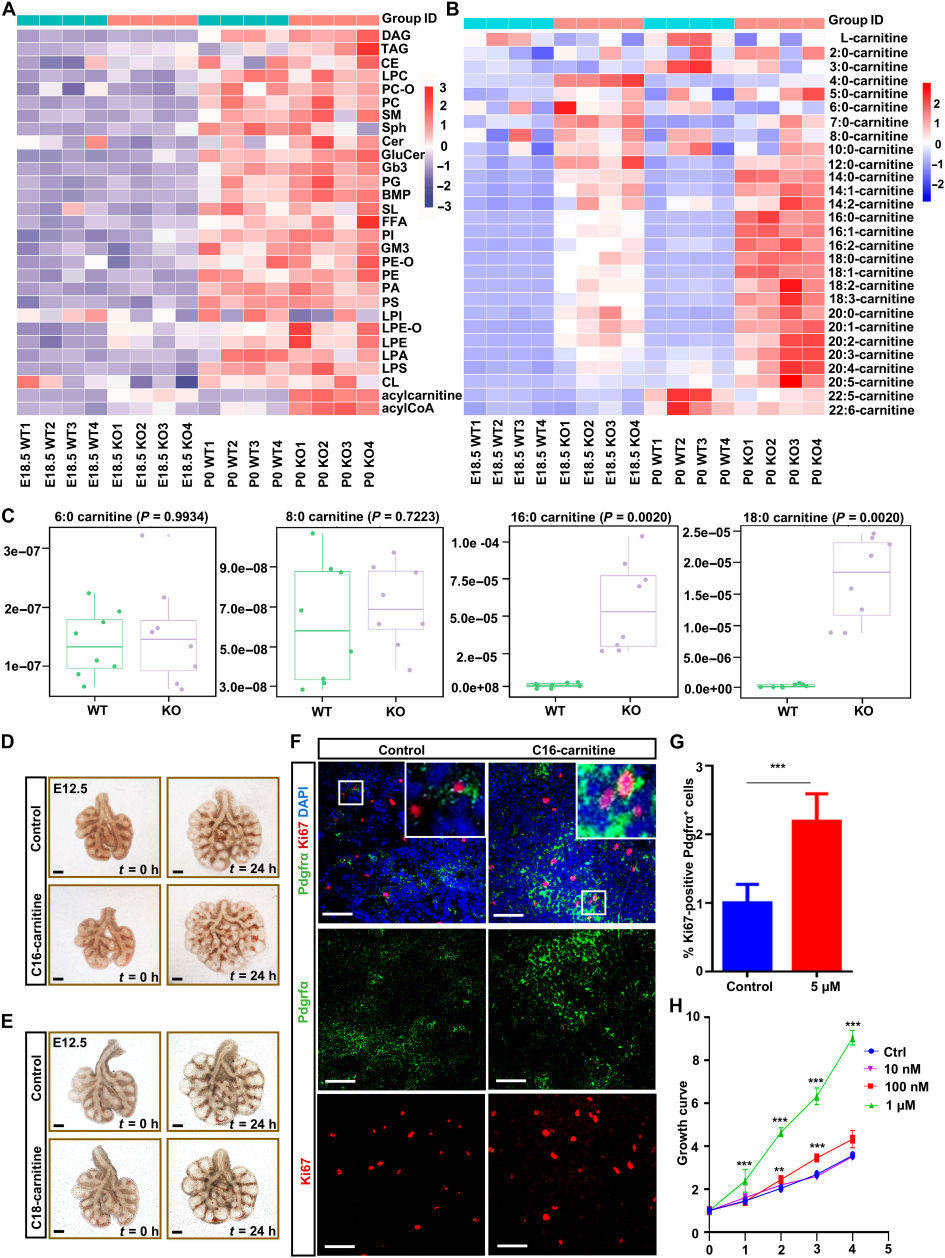
C16-carnitine promoted the proliferation of Pdgfrα^+^ cells. (A) The heatmaps demonstrate the overall alterations in the quantified lipid classes of the control and *Cact^−/−^* lung lipidome at E18.5 and P0. (B) Heatmaps depicting the comprehensive changes in the quantitation of individual acylcarnitine species within the entire lung lipidome at E18.5 and P0 are presented. (A and B) *Z* scores of lipid levels, expressed as molar fractions normalized to total polar lipids (MFP), were plotted for 4 independent animals (*n* = 4) at each developmental stage. (C) The box plots illustrate the maximum change in acylcarnitines throughout lung development, with the magnitude of the *P* value serving as a basis for this representation. *n* = 4 independent animals for per group. Changes were compared using ANOVA. (D and E) Images of lung explants cultured with the vehicle, C16-carnitine, or C18-acylcarnitine. *n* = 8 independent animals. Scale bars: 200 μm. (F) Immunofluorescence stain of Pdgfrα and Ki67 on lung explants cultured with the vehicle, C16-carnitine. Scale bars: 50 μm. (G) The ratio of Ki67 and Pdgfrα double-positive cells to Pdgfrα-positive cells was quantified. *n* = 9 per genotype were used for this quantification. (H) Growth curve analysis of primary mesenchymal cells treated with the vehicle and C16-carnitine derived from lung tissue. (G and H) The data are presented as mean ± SEM.***P < 0.001 (Student *t* test). WT, wild-type mice; KO, knockout mice.

To investigate the molecular mechanism involved in mesenchymal cell proliferation in *Cact^−/−^* mice, we scrutinized entire lung homogenates from control and *Cact^−/−^* mice at the late embryonic period (E18.5) and P0 by quantitative lipidomics approach. Across all samples, 29 lipid categories were confidently identified, visually represented in a heatmap (Fig. 5A and B and Fig. S7). The analysis revealed a conspicuous accumulation of acylcarnitines in *Cact* knockout samples. Notably, the abnormal acylcarnitine patterns suggested increased amounts of long-chain acylcarnitines, especially C16-acylcarnitines and C18-acylcarnitines (Fig. 5B and C).

### C16-acylcarnitine activates mesenchymal cell proliferation

To investigate the potential involvement of C16-acylcarnitine or C18-acylcarnitine in mesenchymal progenitor proliferation, we employed an early embryonic lung organ culture system, enabling controlled manipulation and observation of both epithelial and mesenchymal morphogenesis. Upon treatment with C16-acylcarnitine and C18-acylcarnitine, respectively, the lung explants were subjected to an evaluation of the enhancement in the population density of epithelial and mesenchymal cells over 24 h (Fig. 5D and E).

Intriguingly, when treated with a comparatively low concentration of 5 μM C16-acylcarnitine, the proliferation of mesenchymal cells was activated (Fig. 5F and G). Consistent results were obtained in isolated primary cell lines from wild-type mice, where treatment with 1 μM C16-acylcarnitine significantly increased the number of proliferating Pdgfrα^+^ cells, which further supported that a specific concentration of C16-acylcarnitine indeed promotes the proliferation of mesenchymal cells (Fig. 5H). However, despite subjecting the explants to a range of C18-acylcarnitine concentrations (5 to 500 μM) for 24 h, no discernible abnormalities in proliferation were observed in either mesenchymal or epithelial cells (Fig. S8A and B). The findings indicate that C16-acylcarnitine plays a pivotal role in stimulating the proliferation of mesenchymal cells, thereby contributing to their activation.

### C16-acylcarnitine binds to Samhd1 and promotes the proliferation of mesenchymal cells

With the aim of delving deeper into the molecular processes that support the stimulatory effect of C16-acylcarnitine on mesenchymal cell proliferation, we sought to understand the impact on energy production. Given the involvement of long-chain acylcarnitine in fatty acid β-oxidation, a crucial process in energy production, we first assessed the energy metabolism in *Cact^−/−^* mice. The adenosine monophosphate-activated protein kinase (AMPK), an essential cellular energy sensor within eukaryotes, modulates acetyl-CoA carboxylase (ACC), a meticulously regulated enzyme integral to fatty acid biosynthesis, via both allosteric and phosphorylation modalities [51,52]. Western blotting analysis revealed that in *Cact^−/−^* mice, there was no activation of AMPK phosphorylation and inhibition of ACC (Fig. S8D and E). Given that mitochondria serve as the primary source of cellular energy, predominantly supplying adenosine triphosphate through oxidative phosphorylation, we assessed oxygen consumption rate (OCR) in isolated primary cells from wild-type and mutant mice to determine potential differences in oxidative phosphorylation, finding that mitochondrial function remains unimpaired in *Cact^−/−^* cells, ensuring normal energy production despite the absence of Cact (Fig. S8F).

**Fig. 6.**
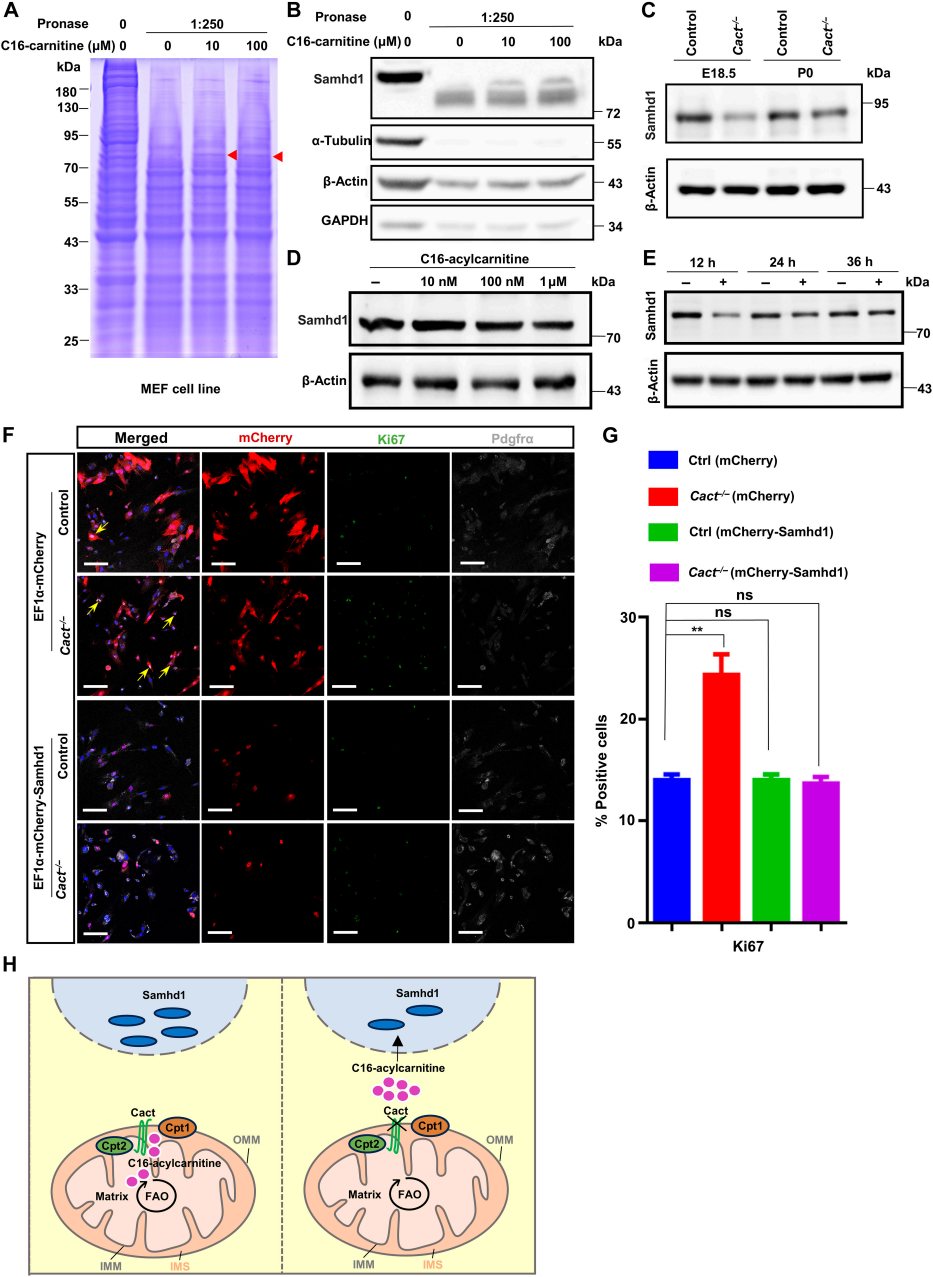
C16-carnitine binds and inhibits sterile alpha motif domain and histidine-aspartate domain-containing protein 1 (Samhd1). (A) Drug affinity responsive target stability (DARTS) has identified Samhd1 as a C16-carnitine-binding protein. The red arrowhead indicates a protected band. (B) DARTS analysis confirms the specific binding interaction of C16-carnitine with Samhd1. (C) The expression level of Samhd1 in control and *Cact^−/−^* lung tissue was detected by western blot. (D) Western blots analysis Samhd1 expression with the treatment of different concentrations of C16-carnitine (10 nM, 100 nM, and 1 μM) in cultured primary mesenchymal cells. The relative protein expression levels are presented in the table above. β-Actin was employed as a loading control. The experiment was conducted on 3 occasions, yielding comparable results on each occasion. (E) Western blots analysis showing decreased Samhd1 expression after 1 μΜ C16-carnitine treated for 12, 24, and 36 h. (F) The images illustrate the representative immunostaining of Ki67 and Pdgfrα in control and *Cact^−/−^* Pdgfrα cells that were transfected with the plasmid of EF1α-mCherry or EF1α-mCherry-Samhd1. The arrowheads indicate the presence of Ki67-positive cells. It is noteworthy that the overexpression of Samhd1 significantly rescued proliferation in *Cact^−/−^* Pdgfrα cells. Scale bars: 100 μm. (G) Statistical diagram of the percentage of Ki67^+^ mesenchymal cells. Data are presented as mean ± SEM. **P < 0.01; ns, not significant (ANOVA). (H) Cact as a key transporter of carnitine–acylcarnitine during fatty acid oxidation (FAO), whose absence leads to noticeable accumulation of long-chain acylcarnitine. C16-carnitine accumulation reduces the protein level of Samhd1 in Pdgfrα^+^ mesenchymal progenitors, leading to a remarkable increase in proliferation level. Meanwhile, the morphology maintenance of alveolar type I cells is impaired, leading to defects in lung development. GAPDH, glyceraldehyde-3-phosphate dehydrogenase; MEF, mouse embryonic fibroblast; OMM, outer mitochondrial membrane; IMM, inner mitochondrial membrane; IMS, intermembrane space.

Given the intricate interplay between many of these regulatory pathways and lipid metabolism, we sought to elucidate the function of C16-acylcarnitine in cell signaling. Employing the drug affinity responsive target stability (DARTS) technique, a potent target screening approach, we aimed to enhance the discovery of C16-acylcarnitine targets. Considering that pivotal targets of C16-acylcarnitine are probably evolutionarily preserved, we utilized mouse embryonic fibroblasts (MEFs) as a readily cultivable protein source for DARTS (Fig. 6A and Fig. S8G). Comprehensive mass spectrometry (MS) assessment pinpointed deoxynucleoside triphosphate triphosphohydrolase Samhd1 (comprising the SAM and HD domains, protein 1) as one of the most prevalent and prominent proteins found in the sample treated with C16-acylcarnitine. The interaction between C16-acylcarnitine and Samhd1 was confirmed using western blotting (Fig. 6B). Notably, Samhd1 protein levels were indeed reduced in *Cact^−/−^* cells (Fig. 6C and Fig. S8H), and C16-acylcarnitine inhibiting the expression of Samhd1 was consistently observed in MEF cells (Fig. 6D and E and Fig. S8I and J). We proceeded to investigate whether the elevated expression of Samhd1 in *Cact* mutants contributes to the defects observed in mesenchymal cells. Overexpressing Samhd1 in isolated primary cells obtained from *Cact* mutant lungs resulted in a significant reduction in proliferation (Fig. 6F and G). This implies that diminished Samhd1 contributes to the proliferation abnormalities observed in lung mesenchymal cells of *Cact^−/−^* mice. It is now clear that Samhd1 is a major regulator of the DNA precursor pool in mammalian cells and modulates cell proliferation by participating in the regulation of cellular deoxynucleoside triphosphate concentration [53].

## Discussion

In this research, we elucidated an intriguing metabolic cascade where Cact-facilitated C16-acylcarnitine binds to the Samhd1 protein, eliciting a marked decrease in its abundance. This intricate interaction subsequently modulates the proliferation of lung mesenchymal progenitors, a pivotal process that underpins the development of respiratory distress, thereby shedding light on a fundamental regulatory mechanism (Fig. 6H). Collectively, these findings offer fresh perspectives on the pivotal function of mesenchymal Cact in modulating neonatal RDS, contributing to a deeper understanding of this complex condition.

Our study sheds light on a potential association between the depletion of Cact and neonatal RDS, a formidable contributor to hospitalization rates and mortality among young children, thereby emphasizing the urgency for further exploration of this link and its implications for clinical management [54]. While our results do not exclude the possibility of *Cact* absence contributing to heart or liver defects, it is notable that both conventional knockout and mesenchymal-specific conditional knockout of *Cact* in pups display elevated C16-acylcarnitine levels (Fig. 5). This leads to alveolar collapse and death shortly after birth, showing symptoms consistent with clinical neonatal respiratory distress (Fig. 3A). These results provided direct evidence that the accumulation of C16-acylcarnitine in mesenchymal cells is the root cause of lung developmental defects. Consistent with our results, CACTD patients presented with respiratory distress, who died in the neonatal period, along with an increased (C16-acylcarnitine + C18-acylcarnitine)/C0-acylcarnitine ratio [38,39,55,56]. Thus, our results reveal the pathological cause underlying neonatal RDS. Here, we propose that restricting CACTD patients’ diet from their usual eating patterns, such as providing practical guidance for low-C16-acylcarnitine intake and addressing dehydration, may be beneficial for their recovery and improvement. From our observation regarding the lack of impact on lung development in normal embryos, despite the down-regulation of Cact expression and subsequent increase in C16-carnitine levels at late embryonic stages, we propose the following explanations to address this phenomenon: First, the canalicular stage transitions to the alveolar stage at E18.5 during lung development, which is accompanied by the determination of cell fate and the activation of cellular activities, ultimately leading to the formation of structurally complete lung architecture. Meanwhile, the expression of Cact decreases during this transition, and the observed correlation between the 2 implies that Cact and C16-carnitine may play a role in normal lung development. In addition, in the later stages of embryonic development, the increase in C16-carnitine levels does not lead to any apparent defects, likely because the dynamic changes in the levels of Cact and C16-carnitine remain within the physiological range. This is different from the changes caused by Cact deletion, in both time and amplitude. Consequently, it is reasonable that the defects in *Cact*-knockout mice would not manifest during the later stages of normal embryonic development.

Our findings offer cellular-level evidence implicating CACTD in the pathogenesis of neonatal RDS. *Cact* mutant lungs display defects in (a) mesenchymal cell proliferation during lung alveologenesis and (b) ATI cells’ morphogenesis during the saccular stage. Consistent with previous reports, excessive proliferation of mesenchymal cells results in heightened lung tissue density, precipitating pulmonary developmental defects and lung pathologies [57]. An impairment in the development of ATI cells, crucial for facilitating gas exchange, may lead to neonatal respiratory distress [58,59]. Pulmonary mesenchymal-specific knockout *Cact* in mice suggests that morphological damage to ATI cells is attributable to mesenchymal cell proliferation, a notion supported by numerous studies [50,60–63].

In our study, we observed an intriguing phenomenon where mesenchymal cells exhibiting elevated levels of C16-acylcarnitine demonstrated minimal alterations in their energy metabolism. This finding is particularly noteworthy given the established role of acylcarnitines, including C16-acylcarnitine, in facilitating the transport of fatty acids into mitochondria for β-oxidation, a process crucial for energy production. Several potential explanations could account for this phenomenon: Firstly, mesenchymal cells exhibit metabolic adaptability, enabling them to reprogram their energy metabolism to maintain stability [64,65]. Therefore, despite fluctuations in acylcarnitine levels, mesenchymal cells possess compensatory mechanisms. These mechanisms may include adjustments in fatty acid uptake, modulation of β-oxidation rates, or the activation of alternative energy-generating pathways. Moreover, the energy supply for mesenchymal cells relies on substrates such as glucose and fatty acids [66,67]. It is essential to consider the complexity of cellular metabolism, where multiple pathways and regulatory networks interact to maintain energy homeostasis [64,68]. Thus, changes in one metabolite, such as C16-acylcarnitine, may not necessarily translate into proportional alterations in overall energy metabolism. Instead, these changes could be buffered by other metabolic pathways or regulated by intricate signaling networks within the cell.

Our results indicate that elevated C16-acylcarnitine stimulates mesenchymal cell proliferation (Fig. 5F to H). The accumulation of C16-acylcarnitine did not result in changes to mitochondrial energy metabolism (Fig. S8D to F). Furthermore, DARTS results demonstrate that C16-acylcarnitine binds to Samhd1, which likely functions as a signaling molecule (Fig. 6A and B). So far, more and more studies described that alteration of FAO metabolite flux can regulate a pathway switch to determine lineage specification and stem cell fate [22,29,36]. Succinic acid acts as a signaling molecule that modulates cellular oxidative stress and inflammatory responses [69]. α-Ketoglutarate engages in epigenetic regulation to modulate post-injury cardiac regeneration and repair [36]. Our results indicate that during mesenchymal cell proliferation, C16-acylcarnitine acts as a signaling molecule by binding to and inhibiting Samhd1, thereby linking metabolic regulation to gene expression modulation. Although we currently lack understanding of how C16-acylcarnitine binds to Samhd1 and modulates its protein levels, it can be speculated that C16-acylcarnitine might influence the stability, localization, or enzymatic activity of Samhd1 [70–74]. In addition, the impact of Samhd1 on proliferation has been extensively reported [75–78].

Overall, our founding establishes that the acylcarnitine metabolic pathway, under the control of *Cact*, is indispensable for lung development. This regulation occurs through the modulation of mesenchymal progenitor cell proliferation by diminishing Samhd1. These groundbreaking discoveries underscore the vital roles of *Cact* and acylcarnitine in lung development, presenting a promising new direction for the development of therapeutic strategies aimed at mitigating or preventing diseases that are intimately tied to neonatal RDS.

## Materials and Methods

### Animals

GemPharmatech Co., Ltd. produced the first *Cact* gene knockout mouse line (https://www.gempharmatech.com). *Cact* heterozygous mice (*Cact*^*+/*−^) were produced by mating with *ZP3*-Cre transgenic mice. Homozygous knockout mice (*Cact*^−/−^) were subsequently obtained by intercrossing the heterozygous mice. For this study, both male and female littermates with genotypes *Cact^+/+^*, *Cact*^*+/*−^, and *Cact*^−/−^ at embryonic stages E14.5, E16.5, E18.5, and P0 were used. The *Cact^+/+^* littermates served as control animals. To generate lung-specific *Cact* knockout mice (*Shh;Cact*), mice carrying floxed *Cact* alleles (*Cact^fl/fl^*) were crossed with those expressing Cre recombinase under the control of the Shh promoter (*Shh^cre/+^*). The *Shh^cre/+^* allele was previously described [49]. The lung mesenchyme-specific *Cact* knockout mice (*Dermo1;Cact*) were generated by crossing *Cact^fl/fl^* mice with *Dermo1^cre/+^* mice; the *Dermo1^cre/+^* allele was generated as outlined in prior descriptions [79]. Rosa-mTmG reporter mice was generated as previously described [49]. In this experiment, male rodents within the age range of 2 to 8 months and female rodents within the age range of 2 to 4 months were chosen for mating in enclosures at a 1:3 ratio daily between approximately 1700 and 1900. The males were removed prior to 9:00 AM the subsequent day, and a vaginal smear test was conducted on the females to confirm mating at 12:00 AM. On the day of a positive vaginal plug detection, E0.5 was designated as the commencement of the embryonic stage. The mice utilized in this research were inbred on a C57BL/6 genetic background and housed in specific-pathogen-free conditions, maintained within a controlled environment with a regulated temperature and humidity, and subjected to a 12-h light/dark cycle. All procedures involving animals adhered to the protocols approved by the animal welfare committees of the Institute of Genetics and Developmental Biology, Chinese Academy of Sciences. Furthermore, all animal studies conducted were in strict compliance with pertinent ethical standards and guidelines.

### Lipidomics

#### Lipid extraction

Lipids were extracted from lung tissues using the Bligh and Dyer protocol as reported [80]. In brief, 900 μl of a solution containing chloroform and methanol in a 1:2 ratio, supplemented with 10% deionized water, was added to the samples. The samples were then homogenized using an automated bead mill (OMNI, Seattle, WA, USA) at a speed of 5 m/s for 8 s, with 2 cycles and a 5-s pause between each. Following homogenization, the samples were incubated at 4 °C with a rotation speed of 1,500 revolutions per minute for 1 h. After incubation, 400 μl of deionized water and 300 μl of chloroform were introduced to induce phase separation. The resultant lower organic layer was meticulously transferred to a clean tube. A subsequent extraction was performed by adding 500 μl of chloroform. The organic extracts from both extraction rounds were pooled and dried using a SpeedVac system in OH mode. Subsequently, the dried samples were reconstituted in a 1:1 volume ratio of chloroform to methanol for lipidomic analysis [19].

#### MS analyses

Quantitative analysis of the lung lipidome was conducted using targeted multiple-reaction monitoring techniques. A comprehensive methodology for this approach was recently published in detail [81]. The extracted phospholipids and sphingolipids were analyzed on an Exion ultrahigh-performance liquid chromatography system interfaced with Sciex QTRAP 6500 Plus, utilizing electrospray ionization in both positive and negative ionization modes. A single injection in negative mode was employed to assess phosphatidylethanolamine (PE), PG, phosphatidylinositol (PI), phosphatidic acid (PA), phosphatidylserine (PS), cardiolipin (CL), GM3, sphingomyelin (SM), FFAs, LPE, lysophosphatidylinositol (LPI), lysophosphatidic acid (LPA), lysophosphatidylserine (LPS), and phosphatidylcholine (PC), with specific transitions focused on fatty acyl groups. Additionally, 2 injections in positive mode were conducted to cover PC, lysophosphatidylcholine (LPC), SM, ceramide, GluCer, lactosylceramide (LacCer), and sphingosine (Sph), ensuring that all detected lipids resided within the linear range of the mass spectrometer’s detection capabilities. The instrumental settings were configured as follows: curtain gas flow rate set to 20, temperature maintained at 400 °C, and GS1 and GS2 both adjusted to 20. Separation of phospholipids and sphingolipids was achieved on a TUP HB silica column with dimensions of 150 × 3 mm and a particle size of 3 μm. Mobile phase A comprised a mixture of chloroform, methanol, and aqueous ammonia in the proportion 895:100:5 (v/v/v), while mobile phase B consisted of chloroform, methanol, water, and aqueous ammonia in the ratio 270:650:70:10 (v/v/v/v). For the quantification of lipids, internal standards such as d9-PC32:0(16:0/16:0), dimyristoyl-phosphatidylcholine, d9-PC36:1p(18:0p/18:1), d7-PE33:1(15:0/18:1), PE14:0/14:0, d9-PE36:1p(18:0p/18:1), d31-PS(16:0/18:1), d7-PG33:1(15:0/18:1), PG14:0/14:0, d7-PI33:1(15:0/18:1), d7PA33:1(15:0/18:1), PA 17:0/17:0, bis(monoacylglycero)phosphate 14:0/14:0, d5-CL 72:8(18:2)4, d8-SM d18:1/18:1, SM d18:1/12:0, ceramide d18:1/d7-15:0, ceramide d18:1/17:0, GluCer d18:1/8:0, d3-LacCer d18:1/16:0, GM3 d18:1/18:0-d3, Gb3-d18:1/17:0, SL-d18:1/17:0, d7-LPC 18:1, LPC C17:0, d7-LPE 18:1, LPE C17:0, LPA-C17:0, LPI-C17:1, LPS-C17:1, LPG-C17:1, S1P d17:1, and Sph-d17 were sourced from Avanti Polar Lipids. Additionally, d3-16:0-carnitine was obtained from Cambridge Isotope Laboratories, while d31-FFA-16:0 and d8-FFA-20:4 were procured from Sigma-Aldrich and Cayman Chemicals, respectively. The quantification of neutral lipids, including TAGs and diacylglycerols (DAGs), was performed using a modified reversed-phase high-performance liquid chromatography (HPLC)/multiple-reaction monitoring technique on an Agilent 1260 HPLC system interfaced with a SCIEX QTRAP 5500 mass spectrometer, operating under positive electrospray ionization mode [82]. Neutral lipids underwent separation on a Phenomenex Kinetex C18 column (dimensions: 4.6 × 100 mm; particle size: 2.6 μm) employing an isocratic elution system consisting of chloroform, methanol, and 0.1 M ammonium acetate in the ratio 100:100:4 (v/v/v), with a flow rate of 300 μl/min. The MS source was configured with the following parameters: a curtain gas flow rate of 10, a temperature maintained at 350 °C, and both nebulizing gases (GS1 and GS2) adjusted to 35. Quantification of short/medium/long-chain TAGs was conducted by comparing their signals to those of spiked internal standards: TAG (14:0)_3_-d5, TAG (16:0)_3_-d_5_, and TAG (18:0)_3_-d_5_ from CDN Isotopes. DAGs were quantified using the internal standards DAG (16:0/16:0)-d_5_ and DAG (18:1/18:1)-d_5_ from Avanti Polar Lipids. The determination of free cholesterol and total cholesteryl esters was carried out through atmospheric pressure chemical ionization in positive ion mode utilizing an Agilent 1260 HPLC system interfaced with a SCIEX QTRAP 5500 mass spectrometer, as previously described [83]. The aqueous polar supernatant obtained from the Bligh and Dyer extraction procedure was directed for the quantification of short-chain (polar) carnitines and CoAs using a Thermo Fisher DGLC U3000 system interfaced with Sciex QTRAP 6500 Plus, according to the previously published methodology [19,84].

### Lung explant culture

Wild-type E12.5 lung tissue was harvested and placed on a nuclear pore-tracking etching membrane (Whatman, 110614). Subsequently, the lungs were cultured at the air/liquid interface in a medium comprising Dulbecco’s modified Eagle medium (DMEM)–F12 (Invitrogen, C11330500BT) and 10% fetal bovine serum (Invitrogen, 16000-044). The lungs were harvested and subjected to analysis following a fixed period of incubation in an incubator at 37 °C and 5% CO_2_ concentration. All lung explant cultures were repeated 3 to 5 times, and embryos were obtained from at least 3 pregnant mice.

### Immunofluorescence staining, imaging, and quantification

For immunofluorescence staining, the sections were subjected to a pretreatment with 0.2% Triton X-100 in phosphate-buffered saline for a period of 15 min at room temperature. Following the blocking step, the sections were incubated with a 0.2% dilution of the primary antibody for one night at 4 °C. This was followed by incubation with phosphate-buffered saline–Triton X-100 at 4 °C for one night and incubation with selected secondary antibodies for 1 h. Images were acquired using a Leica TCS SP8 confocal microscope and a Nikon Ti-E&N-STORM super-resolution microscope system. At least 5 mice of each genotype were imaged, and the number of cells in at least 3 different regions in each image was quantified.

### Whole-mount immunofluorescence staining and imaging

The methodology for whole-mount immunofluorescence staining was based on a previously described approach [85]. In conclusion, lungs at varying stages of development were fixed in 4% paraformaldehyde at 4 °C for 1 h and subsequently dehydrated with methanol. The lungs were incubated at 4 °C overnight with antibodies to Sox2 (Santa Cruz Biotechnology, sc-17320, 1:200) and Sox9 (Millipore, AB5535, 1:200). Subsequently, the appropriate secondary antibody was incubated at 4 °C for a period of 12 h. The stained lungs were observed and imaged using a Leica MZ16F stereomicroscope.

### Histologic analysis and the hematoxylin and eosin staining

For histological analysis, lungs from control or CACT mutant mice were fixed overnight at 4 °C in 4% (w/v) paraformaldehyde (Sigma-Aldrich, USA). Following paraffin embedding (Thermo, USA), the tissues were sectioned at a thickness of 5 μm. The procedure for staining was carried out in accordance with a previously described methodology [86]. The sections were then deparaffinized and rehydrated while hematoxylin and eosin staining was conducted. Following a 5-min staining period with Harris hematoxylin solution, the sections were differentiated in 1% acidic alcohol for a period of 2 to 5 s and then rinsed under running water for a minimum of 20 min. Subsequently, the sections were counterstained for 1 min with an eosin–fluorescein solution. Subsequently, the tissue sections were dehydrated, washed, and mounted. The sections were then examined under a microscope.

### Western blotting

The radioimmunoprecipitation assay buffer composition included 150 mM NaCl, 0.1% sodium dodecyl sulfate, 50 mM Tris (pH 7.4), 0.5% sodium deoxycholate, and 1% NP-40. Protein concentrations were quantified utilizing the bicinchoninic acid (BCA) assay. Approximately 40 μg of total protein was then subjected to standard western blotting, following a protocol previously detailed [87]. The following antibodies were used: anti-Cpt1 (Proteintech, 15184-1-AP, 1:1,000), anti-Cpt2 (Proteintech, 26555-1-AP, 1:1,000), anti-Cact (Proteintech, 15170-1-AP, 1:1,000), anti-Pdgfrα (Cell Signaling Technology, 3174S, 1:2,000), anti-alpha smooth muscle actin (Santa Cruz Biotechnology, clone sc-32251, 1:5,000), anti-AMPK (Cell Signaling Technology, 2532S, 1:5,000), anti-phosphorylated AMP-activated protein kinase (Cell Signaling Technology, 2535S, 1:5,000), anti-ACC (Cell Signaling Technology, 3662, 1:5,000), anti-phosphorylated ACC (Cell Signaling Technology, 3661, 1:5,000), anti-Samhd1 (Proteintech, 12586-1-AP, 1:1,000), and anti-β-actin (Sigma, A5060, 1:5,000).

### The identification of targets utilizing DARTS

For unbiased target identification, lysis of MEFs was achieved by employing M-PER (Thermo Scientific, 78501), supplemented with protease inhibitor Cocktail Tablets (Roche, 04693116001) and phosphatase inhibitor Cocktail Tablets (Roche, 04906845001). Following the lysis procedure, the lysate was treated with TNC buffer (comprising 50 mM Tris-HCl pH 8.0, 50 mM NaCl, and 10 mM CaCl_2_), after which the protein concentration was determined by means of the BCA Protein Assay Kit (Pierce, 23227). After incubating cell lysates with either the vehicle or C16-acylcarnitine overnight on ice, an additional hour was spent at room temperature before further processing. Digestion by Pronase (Roche, 10165921001) was conducted at room temperature for 45 min, followed by the termination of the reaction using an excess of protease inhibitors and the immediate transfer of the sample to ice. The resulting digests were subjected to separation by sodium dodecyl sulfate–polyacrylamide gel electrophoresis and visualization using Coomassie brilliant blue stain. Protein bands displaying intensified staining in the C16-acylcarnitine lane (indicative of potential targets protected from proteolysis through C16-acylcarnitine binding), along with corresponding regions in the control lane, were meticulously excised for further analysis. Subsequently, in-gel tryptic digestion was conducted in accordance with the established protocol, followed by LC–MS/MS analysis.

### Measurement of OCRs

The OCR was determined by utilizing a Seahorse XF-96 analyzer. Cells were plated onto Seahorse XF-96 cell culture microplates at a density of 30,000 cells per well in DMEM enriched with 10% fetal bovine serum and 10 mM glucose. Subsequently, the plates were incubated for 12 h at 37 °C in a humidified atmosphere containing 5% CO_2_. The C16-acylcarnitine or vehicle control (dimethyl sulfoxide) was administered for a period of 1 h. Prior to measurement, the cells were rinsed with unbuffered DMEM medium (pH 7.4, containing 10 mM glucose) and were then maintained in this medium supplemented with the specified concentrations of C16-acylcarnitine. The OCR was recorded 3 times under basal conditions and normalized to the protein concentration for each well. Statistical analyses were performed utilizing the Wave software.

### Statistical analysis and data visualization

Statistical analyses and data visualization were conducted using the R statistical computing environment (version 4.3). A principal component analysis was conducted using the prcomp function, employing singular value decomposition. Time-dependent variations in major lipid abundance were scrutinized employing analysis of variance coupled with Tukey’s honestly significant difference post hoc tests. The results are depicted graphically using bar charts. Pairwise comparisons are indicated with letters, whereby the presence of shared letters indicates the absence of statistically significant differences (*P* < 0.05) between the groups. Heatmap generation was facilitated through the utilization of the pheatmap package. Sample interclustering was performed utilizing functionalities provided by the factoextra package. Furthermore, fuzzy C-means clustering analysis was executed employing the capabilities of the Mfuzz package.

## Data Availability

Codes for the processing and analysis of the lipidomic data, as well as for generating the related figures, are available from https://github.com/lipidall/lung_lipidomics. The lipidomic datasets of lung development process and gene knockout experiments are deposited in the Mendeley database (https://data.mendeley.com/preview/68jgnnj8gv).
