# Supplementary material for: Palmitoyl-carnitine Regulates Lung Development by Promoting Pulmonary Mesenchyme Proliferation
Source: Research (Wash D C). 2025 Mar 18;8:0620. doi: 10.34133/research.0620 (PMC11914330; doi:10.34133/research.0620)
Supplement: Supplementary 1 — Figs. S1 to S8 [file research.0620.f1.zip › Supplementary Information.docx]

**
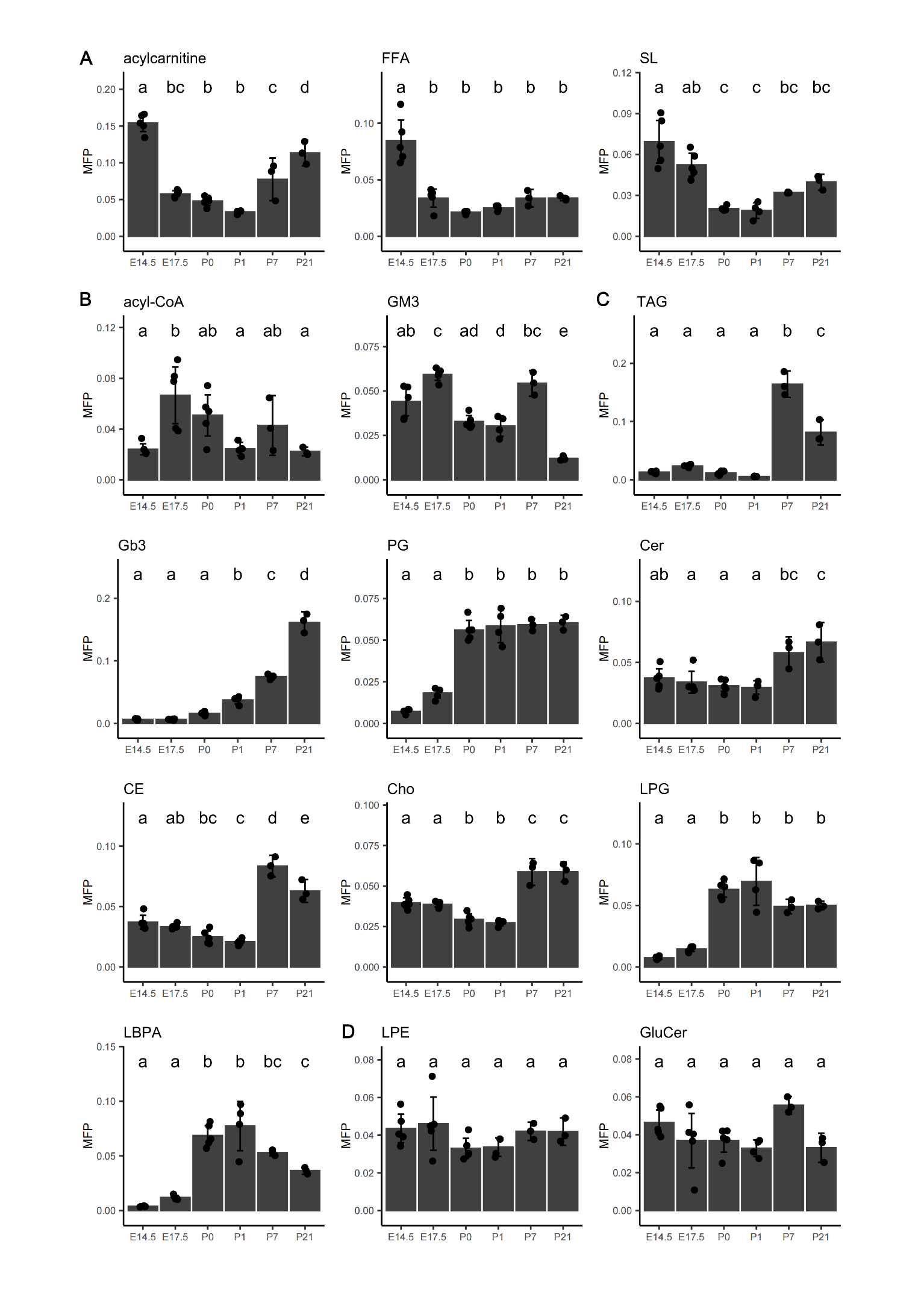
SUPPORTING INFORMATION**

**Figure S1: Temporal changes in lipid classes during lung development.**

**A.** High content of lipids during pseudoglandular stage. **B.** High content of lipids during canalicular stage and saccular stage. **C.** High levels of lipids during alveolar stage. **D.** The content of lipids did not change. MFP: molar fraction of total lipids. Lipid levels were compared across different developmental time points using ANOVA, followed by Tukey's Honest Significant Difference (HSD) post hoc test to determine whether any two groups labeled with a common letter exhibit statistically significant differences at *P* > 0.05.


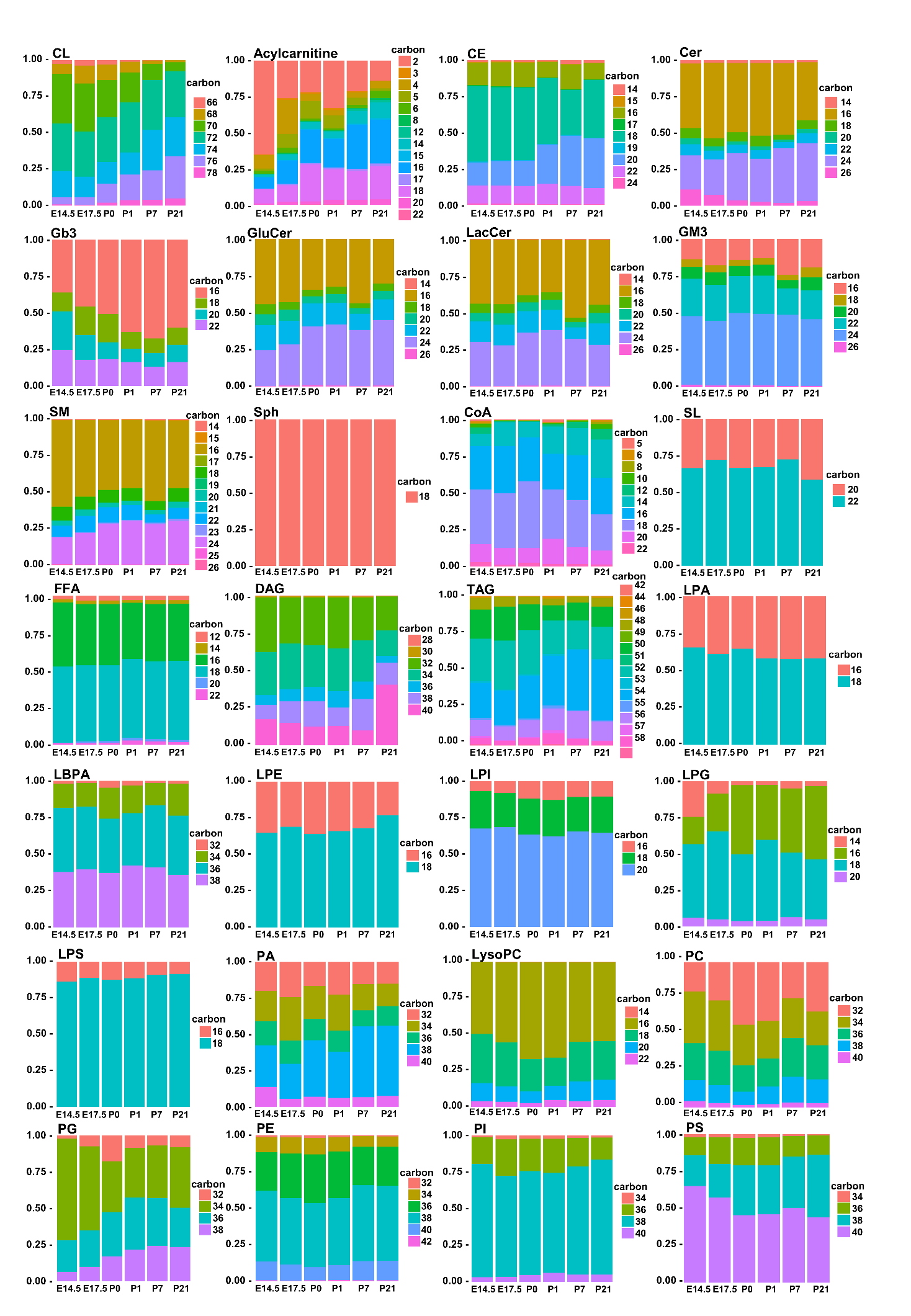


**Figure S2: The length fatty acyl chains of 28 classes of lipid vary during lung development.** Compositional changes in total carbon number of lipids during lung development.


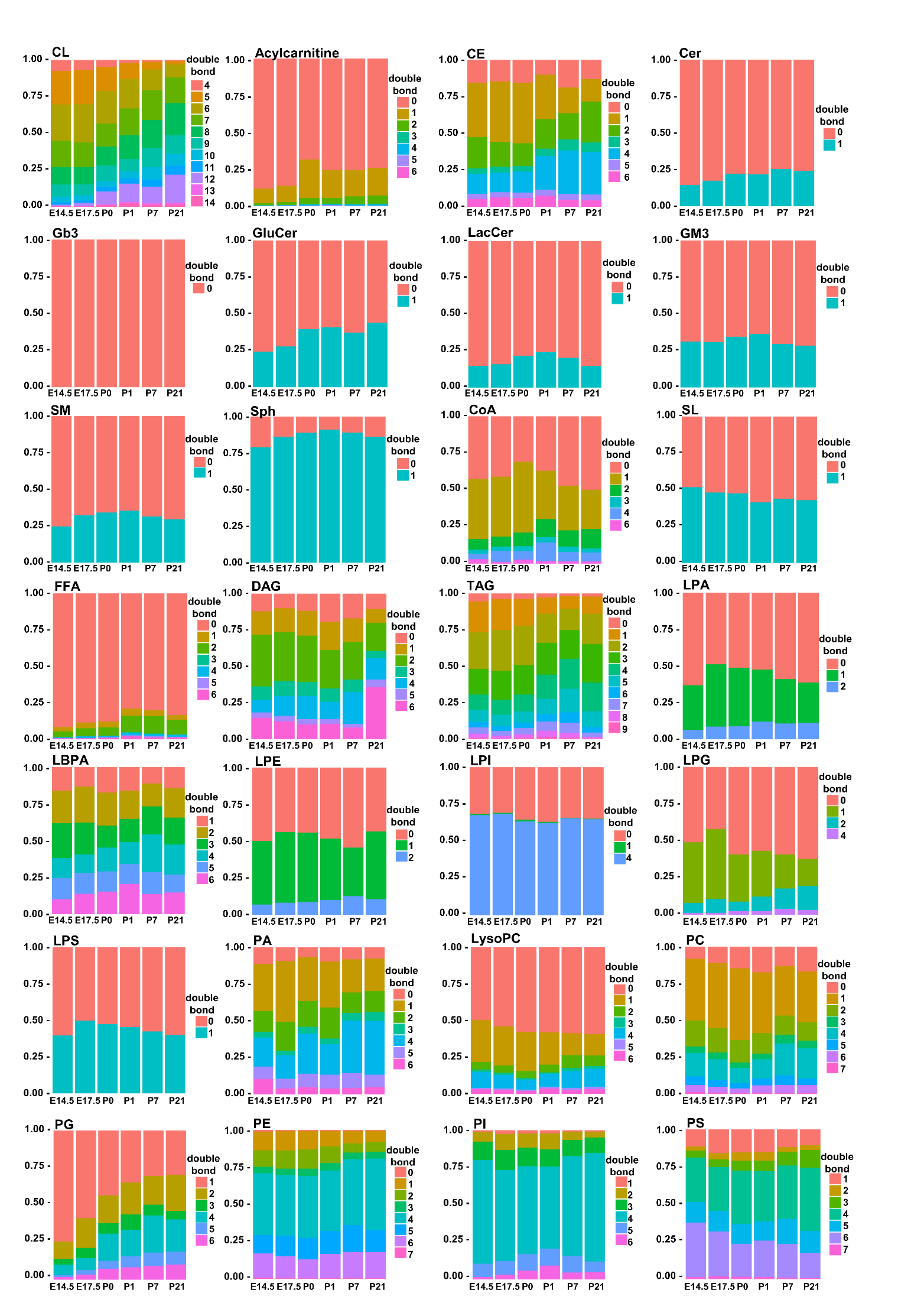


**Figure S3: The saturation of 28 classes of lipid vary during lung development.**

Compositional changes in total double bond number of lipids during lung development.

**
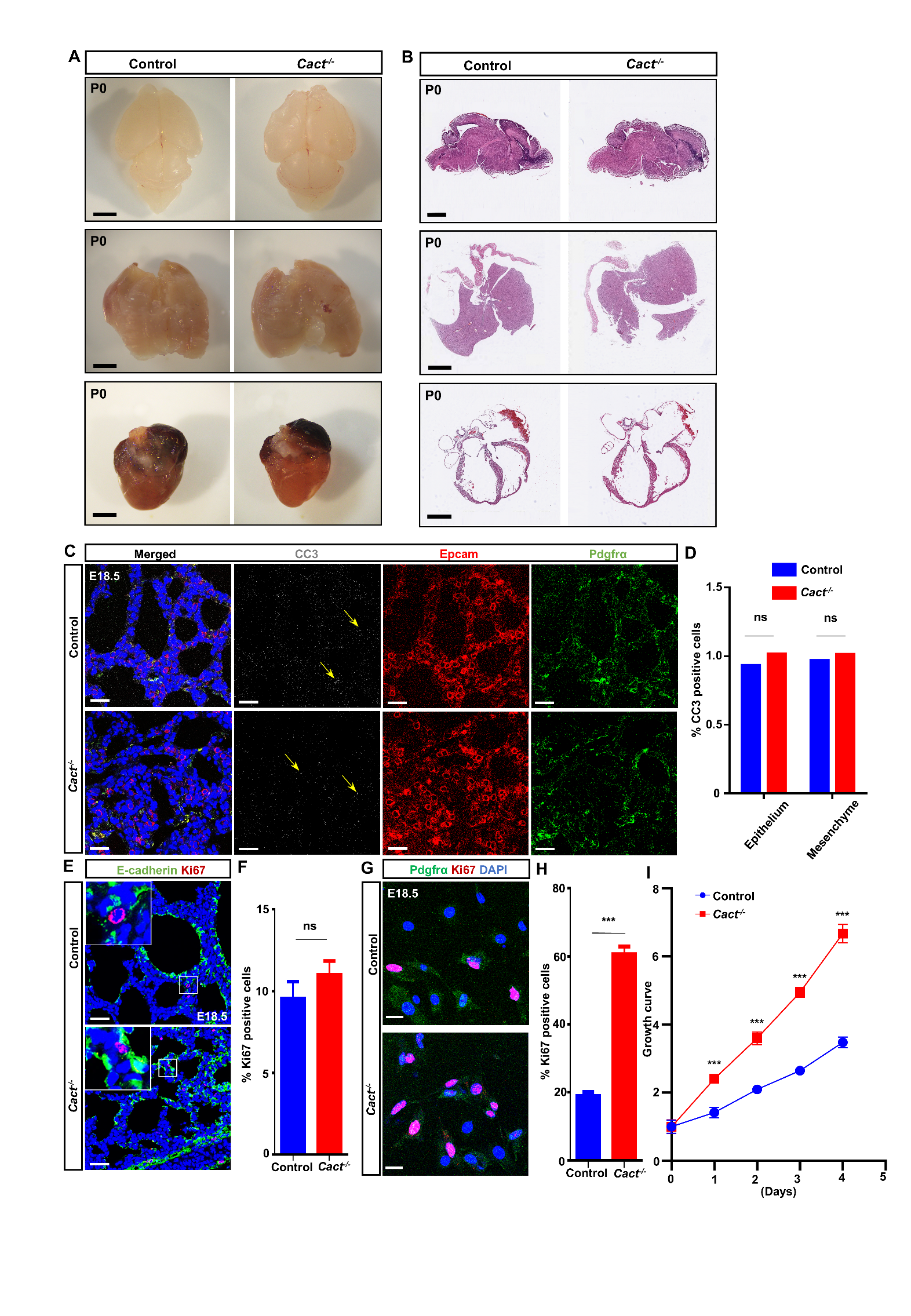
**

**Figure S4: Cact deficiency causes increased proliferation of Pdgfrα^+^ cells.**

**A.** Panoramic anatomical views of the brain, liver, and heart in the control and *Cact^-/-^*. (n = 8). Scale bars: 2 mm. **B.** HE staining presents panoramic views of the brain, liver, and heart in both the control and *Cact^-/-^*. (n = 8). Scale bars: 2 mm. **C.** The following images represent the results of confocal immunostaining for Epcam, Pdgfrα and CC3 on a section of tissue. Arrow points to CC3 positive cells. Scale bars: 20 μm. **D.** The objective is to quantify the number of CC3^+^ cells in E-cadherin^+^ or Pdgfrα^+^ cells. **E.** Fluorescence microscopy analysis of Ki67 and E-cadherin in lung sections form E18.5 mice. Boxed regions are magnified in insets. Scale bars: 50 μm. **F.** Quantification of the number of Ki67^+^ cells in E-cadherin+ cells. **G.** Immunofluorescence staining of Ki67 and Pdgfrα reveals that Pdgfrα^+^ cells proliferation level. The image on the right presents a magnified view of the area delineated by the dashed white boxes. Scale bars: 20 μm. **H.** Quantification of Ki67^+^ cells within the Pdgfrα^+^ cell population. **I.** Growth curve analysis was conducted on control and *Cact^-/-^* cells isolated from lung tissue, with each experiment representing nine biological replicates (n = 9). The data are presented as the mean ± standard error of the mean (s.e.m.). ****P* < 0.001, n.s., not significant. (Student's *t*-test).
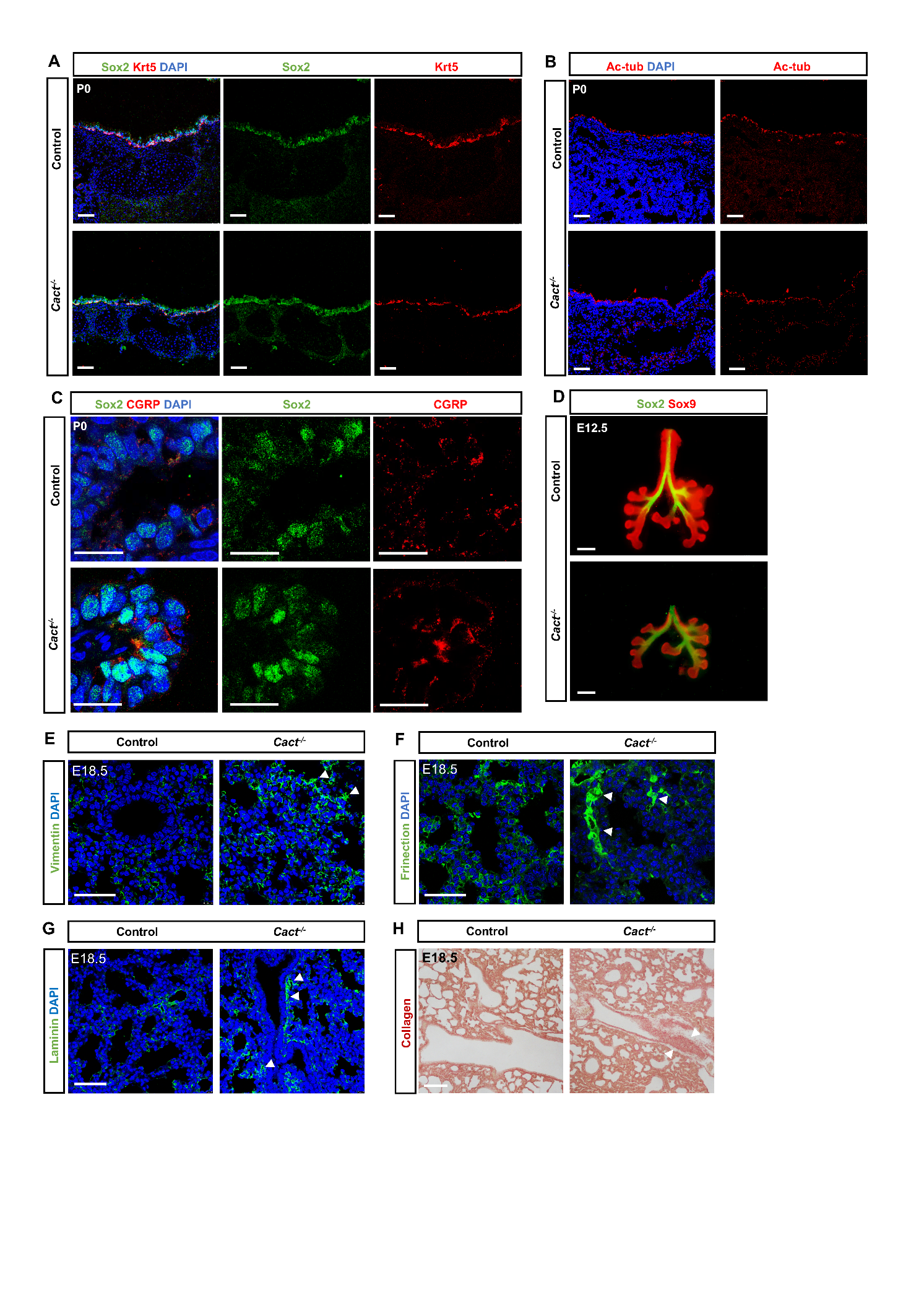


**Figure S5: Absence of Cact has no effect on epithelial cell differentiation.**

**A.** Immunostaining for Sox2 and Krt5 on control (n = 8 mice) and *Cact^-/-^* (n = 8 mice) P0 lung sections. Scale bars: 50 μm. **B.** Immunostain of lung sections from control and *Cact^-/-^* mice with Ac-tub (Acetylated tubulin). Scale bars: 50 μm. **C.** Immunostaining for Sox2 and CGRP in control (n = 8 mice per each stage) and *Cact^-/-^* (n = 8 mice per each stage) lungs at P0. Scale bars: 50 μm. **D.** Whole-mount antibody staining for Sox2 and Sox9 expression in on control (n = 8 mice) and *Cact^-/-^* (n = 8 mice) E12.5 lung. Scale bars: 200 μm. E. Immunostaining for Vimentin on control and *Cact^-/-^* (n = 8 mice) E18.5 lung sections. Scale bars: 50 μm. F. Immunostaining for Frinection on control and *Cact^-/-^* (n = 8 mice) E18.5 lung sections. Scale bars: 50 μm.G. Immunostaining for Laminin on control and *Cact^-/-^* (n = 8 mice) E18.5 lung sections. Scale bars: 50 μm. H. Staining of collagen fibers in lung tissue from day E18.5.


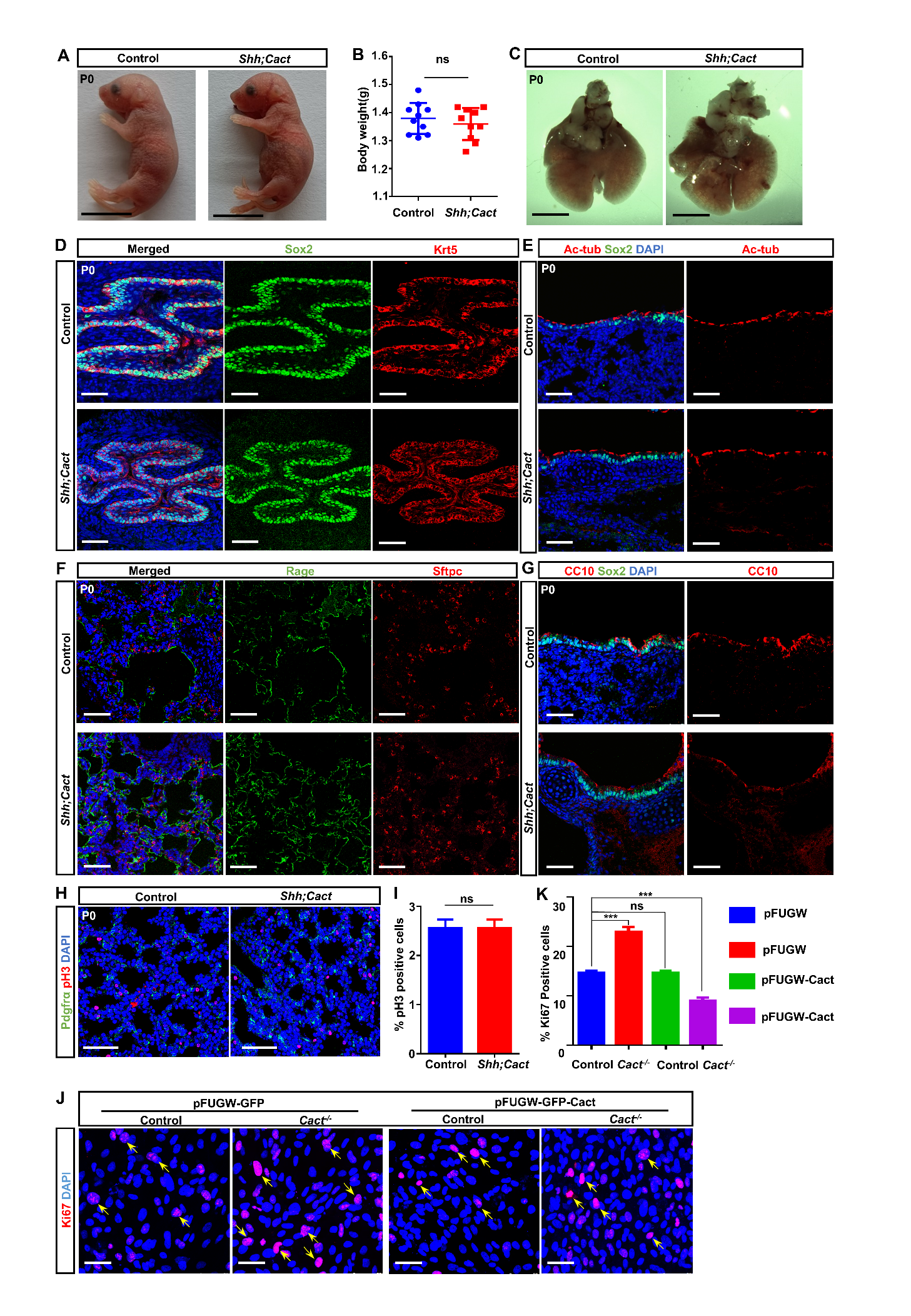


**Figure S6: Epithelial-specific Cact deletion did not impact lung development**

**A.** Gross morphological assessment of P0 mice, comparing control animals (n=10) with *Shh;Cact* transgenic littermates (n=10). Scale bars are set at 1 cm. **B**. Quantitative analysis of body weight at P0 in both control and *Shh;Cact* mutant mice, with a sample size of ten mice per genotype for statistical evaluation. **C**. Ventral visualization of dissected lungs from P0 control and *Shh;Cact* mutant mice. Scale bars: 2.5 mm. **D**. Immunohistochemical staining for Sox2 and Krt5 on lung tissue sections from control (n=8) and *Cact^-/-^* (n=8) P0 mice. Scale bars: 50 μm. **E**. Immunohistochemical staining for acetylated tubulin (Ac-tubulin) in lung sections derived from control and mutant mice. Scale bars :50 μm. **F**. Immunofluorescence staining for Rage and Sftpc on lung sections from control and *Shh;Cact* mice, highlighting alveolar type 1 (AT1) and alveolar type 2 (AT2) cells. Scale bars: 50 μm. **G**. Immunohistochemical staining for Sox2 and CC10 in lungs from control (n=8) and *Cact^-/-^* (n=8) P0 mice. Scale bars: 50 μm. **H**. Immunofluorescence staining for phosphorylated histone H3 (pH3) and platelet-derived growth factor receptor alpha (Pdgfrα) to assess the proliferation level of Pdgfrα^+^ cells. Scale bars: 50 μm. **I**. Quantification of pH3+ cells within the Pdgfrα^+^ cell population. Data are presented as the mean ± standard error of the mean (s.e.m.), with nine biological replicates. Statistical significance was not reached (n.s., not significant) based on Student's *t*-test. **J**. Representative immunostaining images of Ki67 in control and *Cact^-/-^* Pdgfrα cells transfected with pFUGW-GFP or pCMV-GFP-Cact constructs. Arrowheads indicate Ki67-positive cells. Scale bars are 50 micrometers. **K**. Statistical diagram depicting the percentage of Ki67^+^ mesenchymal cells. Data are presented as the mean ± standard error of the mean (SEM) from nine biological replicates. Statistical significance was determined using ANOVA, ***P* < 0.01 and “n.s.” denoting not significant.

**
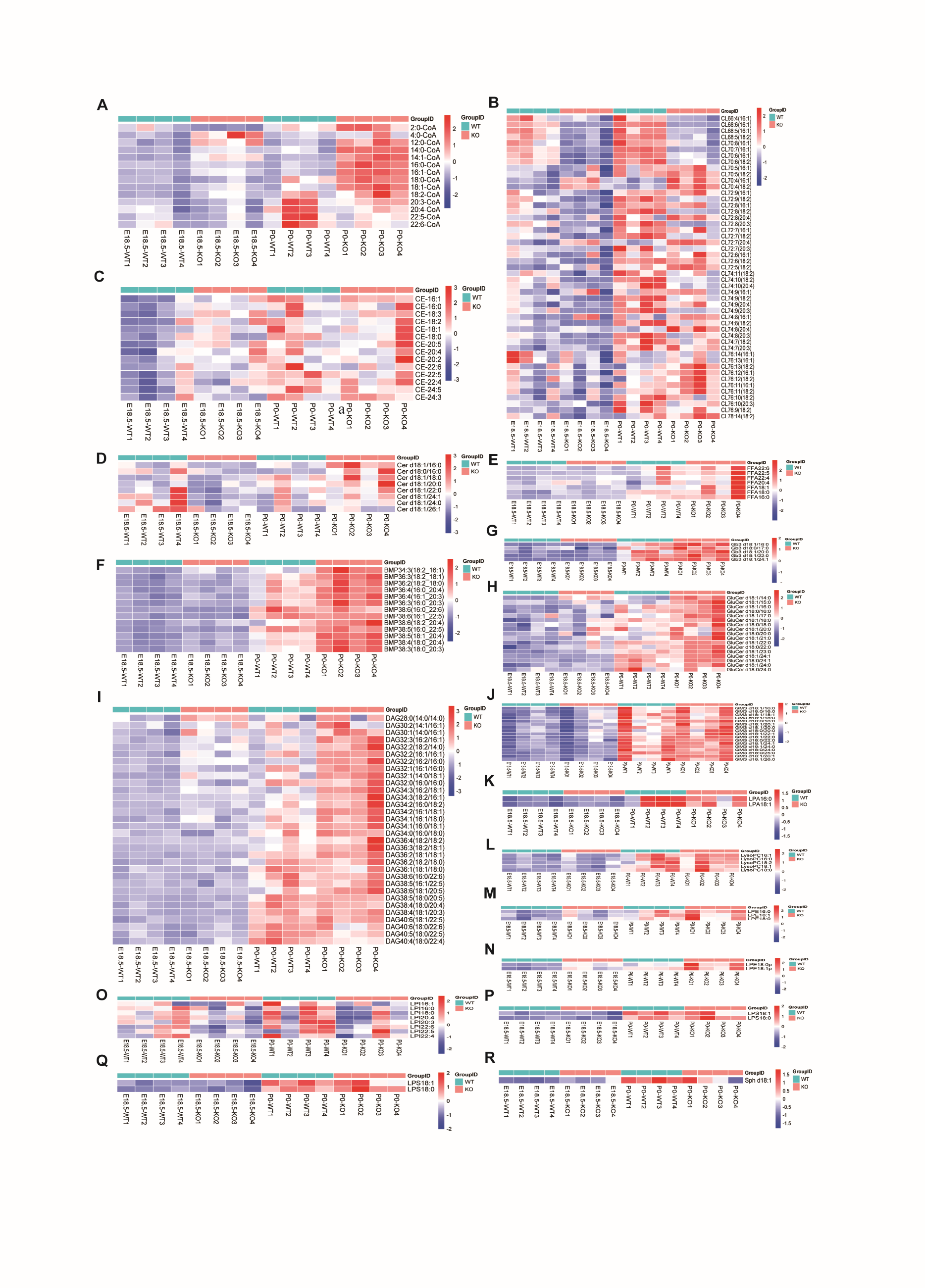
**

**Figure S7: Heatmaps display the comprehensive alterations in quantified lipid class profiles**

Heatmap plots illustrate the normalised lipid abundances (median of replicates) during the process of lung development. The heatmap was subdivided into distinct sections, each corresponding to a specific cluster of lipids.

**
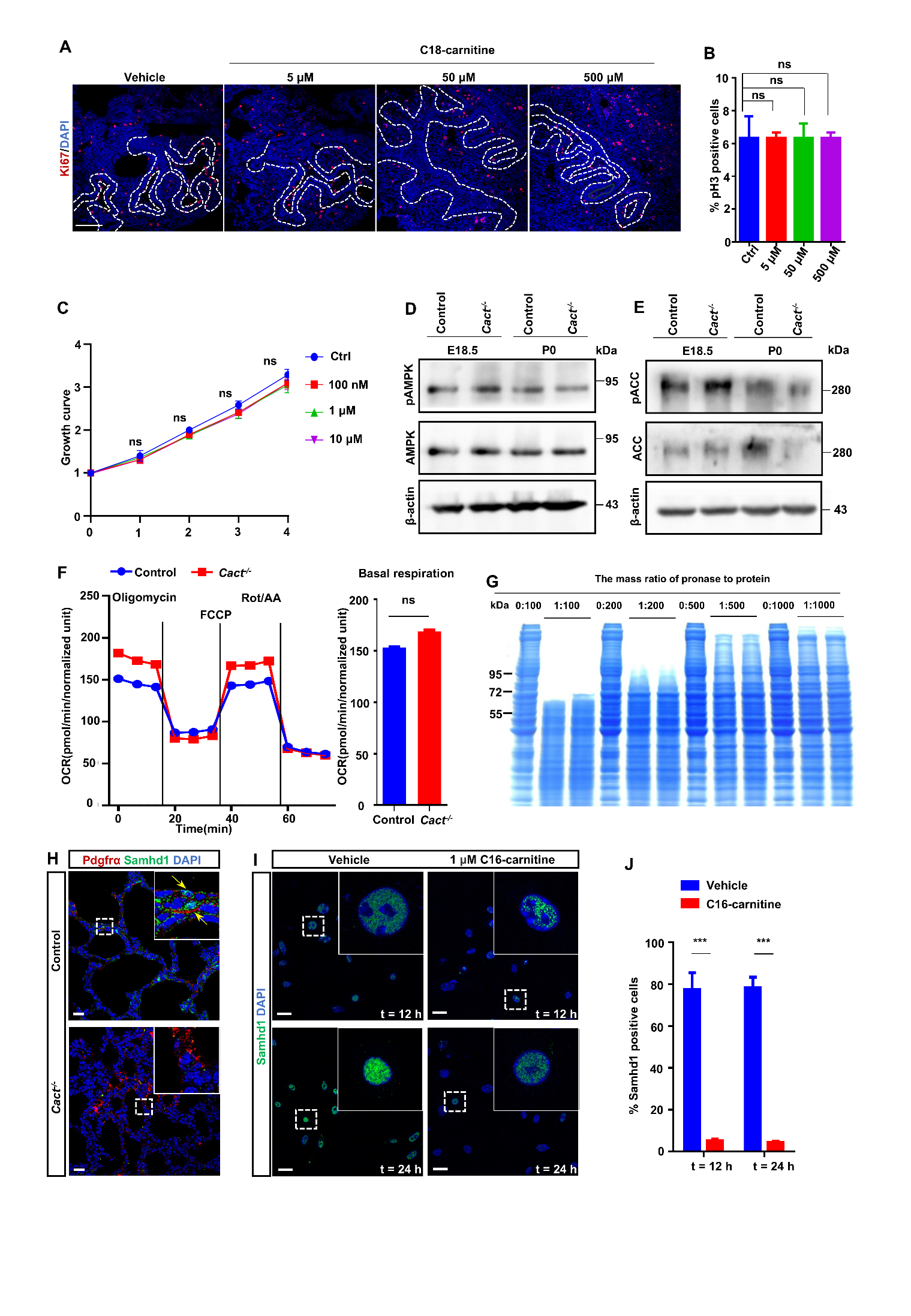
**

**Figure S8: C16-carnitine decreased Samhd1 expression level.**

**A.** Immunofluorescence stain of Pdgfrα and Ki67 on lung explant cultured with vehicle, C16-carnitine. Scale bars: 50 μm. **B.** Quantitative analysis of proliferation in panel a as shown. n = 8 independent animals. Data are presented as the mean ± sem. n.s., not significant. (ANOVA). **C.** Growth curve analysis of primary mesenchymal cells treated with vehicle and C18-acylcarnitine derived from lung tissue. **D-E.** The protein levels of AMPK, pAMPK, ACC and pACC were detected by western blot. **F.** Oxygen consumption rate (OCR) measurements of control and *Cact^-/-^* primary mesenchymal cells. **G.** DARTS-SDS-PAGE results with different ratios of pronase. **H.** Immunostaining for Pdgfrα and Samhd1 in control (n = 8) and *Cact^-/-^* (n = 8) lungs at P0. The images on the right are magnified representations of the regions indicated by the dashed white boxes. Scale bars: 40 μm. **I.** Representative immunostaining images for Samhd1 in vehicle and C16-carnitine treated primary mesenchymal cells. The images on the right are magnified representations of the regions indicated by the dashed white boxes. Scale bars: 25 μm. **J.** Quantification of the number of Samhd1^+^ cells in primary mesenchymal cells. n = 9 biological replicates. Data are represented as mean ± s.e.m. ****P* < 0.001. (Student's *t*-test).
