# Supplementary figures and images for: Palmitoyl-carnitine Regulates Lung Development by Promoting Pulmonary Mesenchyme Proliferation
Source: Research (Wash D C). 2025 Mar 18;8:0620. doi: 10.34133/research.0620 (PMC11914330; doi:10.34133/research.0620)

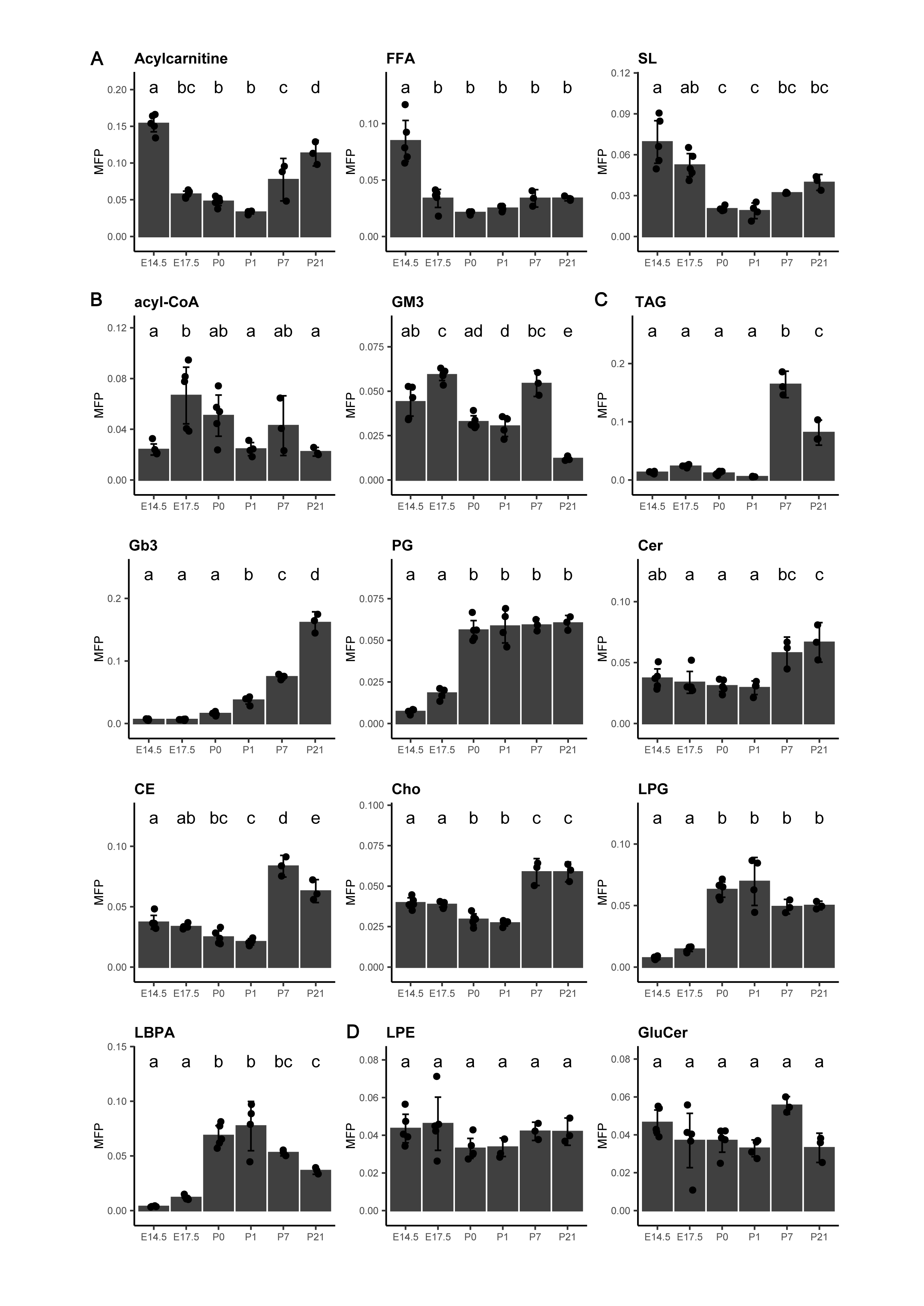

Supplement: Supplementary 1 — Figs. S1 to S8 [file research.0620.f1.zip › Figure S1.tif]

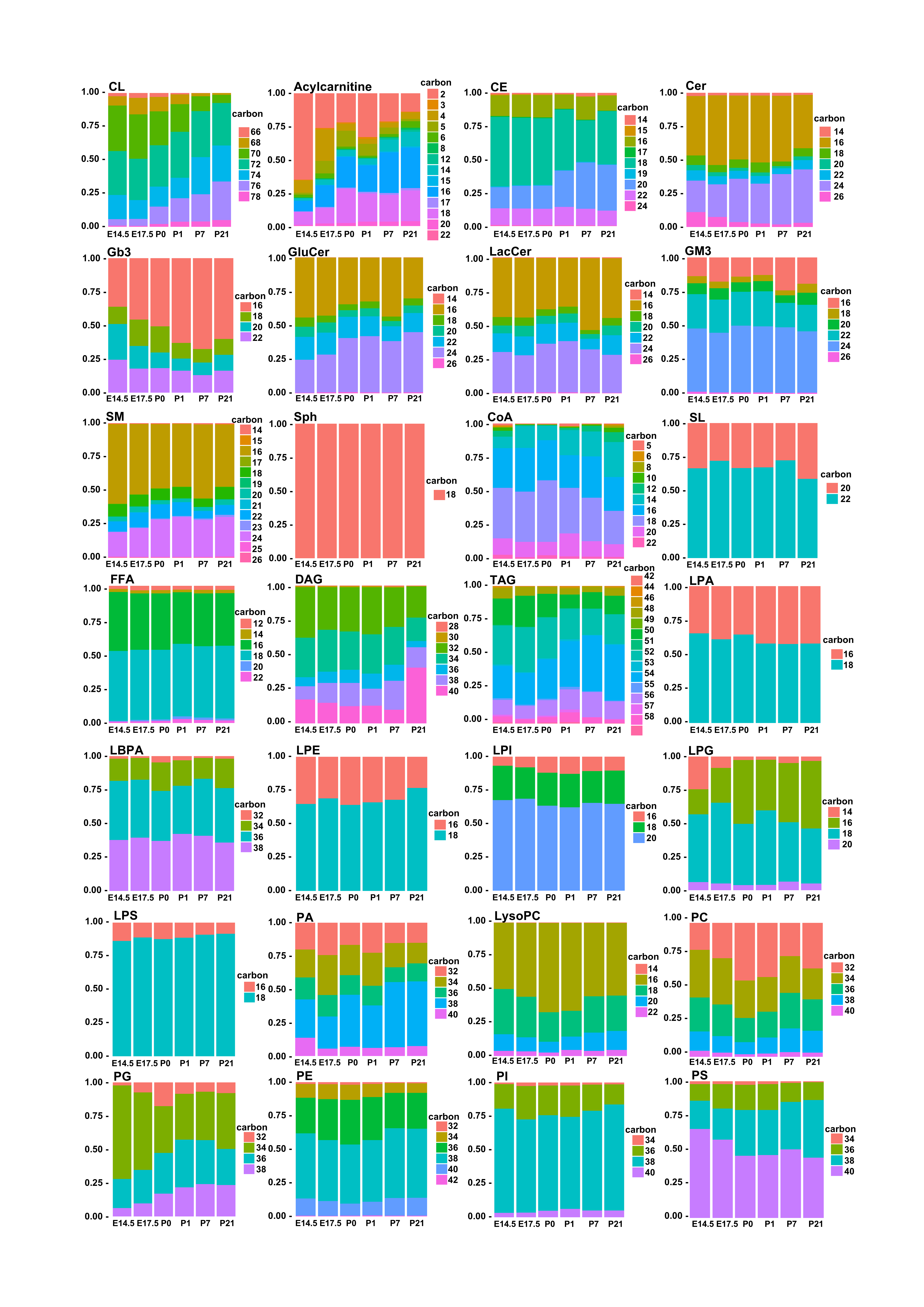

Supplement: Supplementary 1 — Figs. S1 to S8 [file research.0620.f1.zip › Figure S2.TIF]

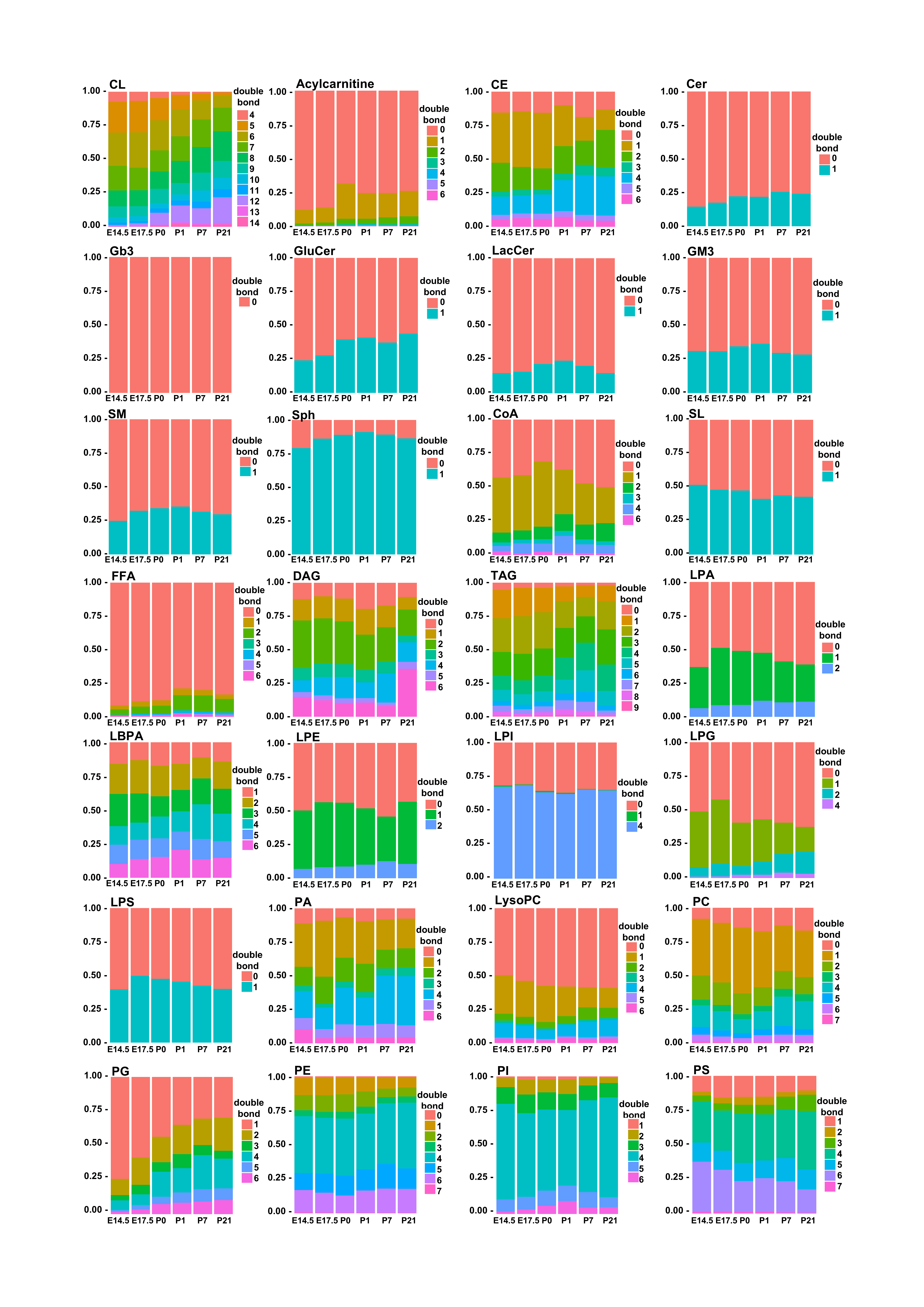

Supplement: Supplementary 1 — Figs. S1 to S8 [file research.0620.f1.zip › Figure S3.TIF]

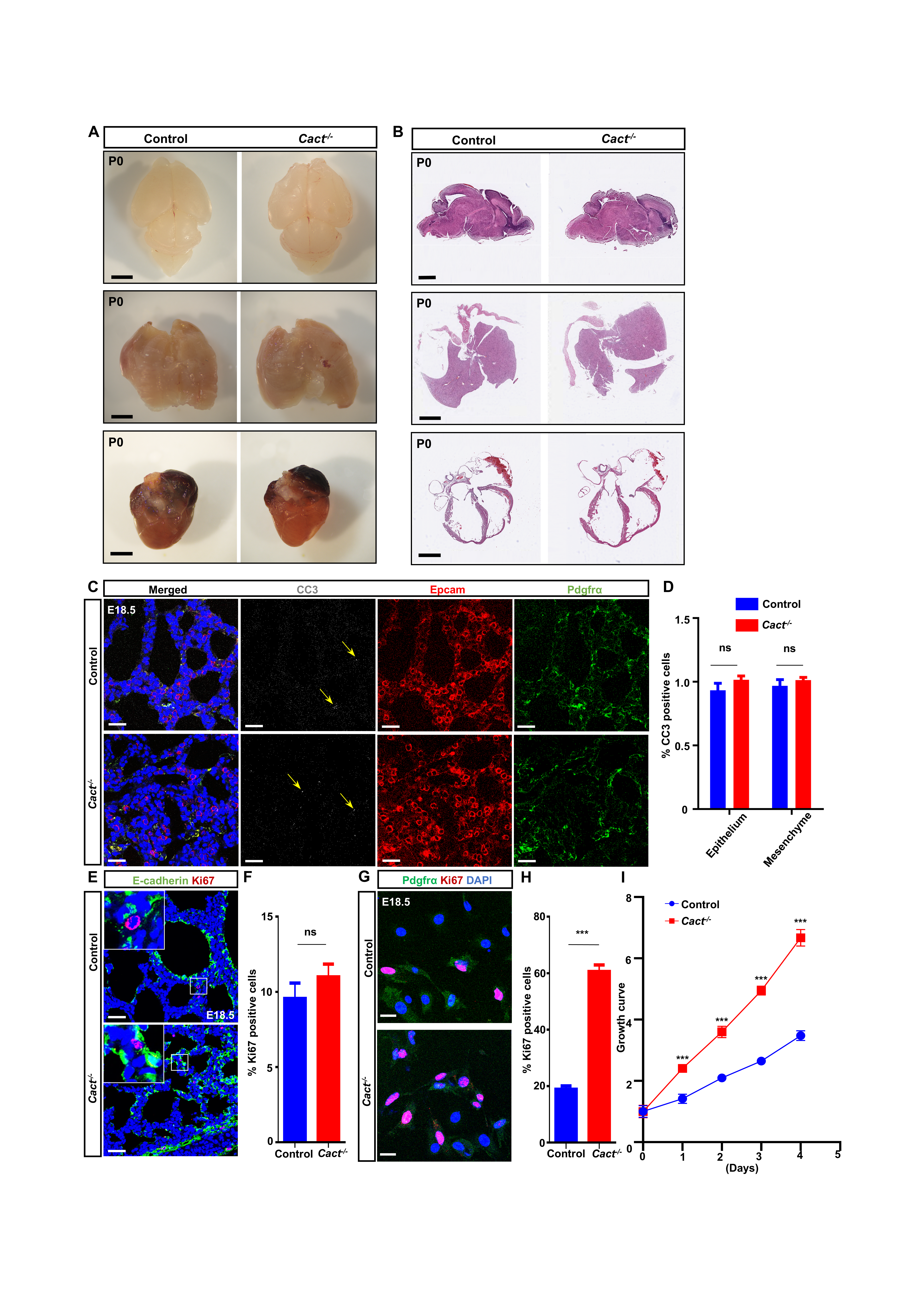

Supplement: Supplementary 1 — Figs. S1 to S8 [file research.0620.f1.zip › Figure S4.TIF]

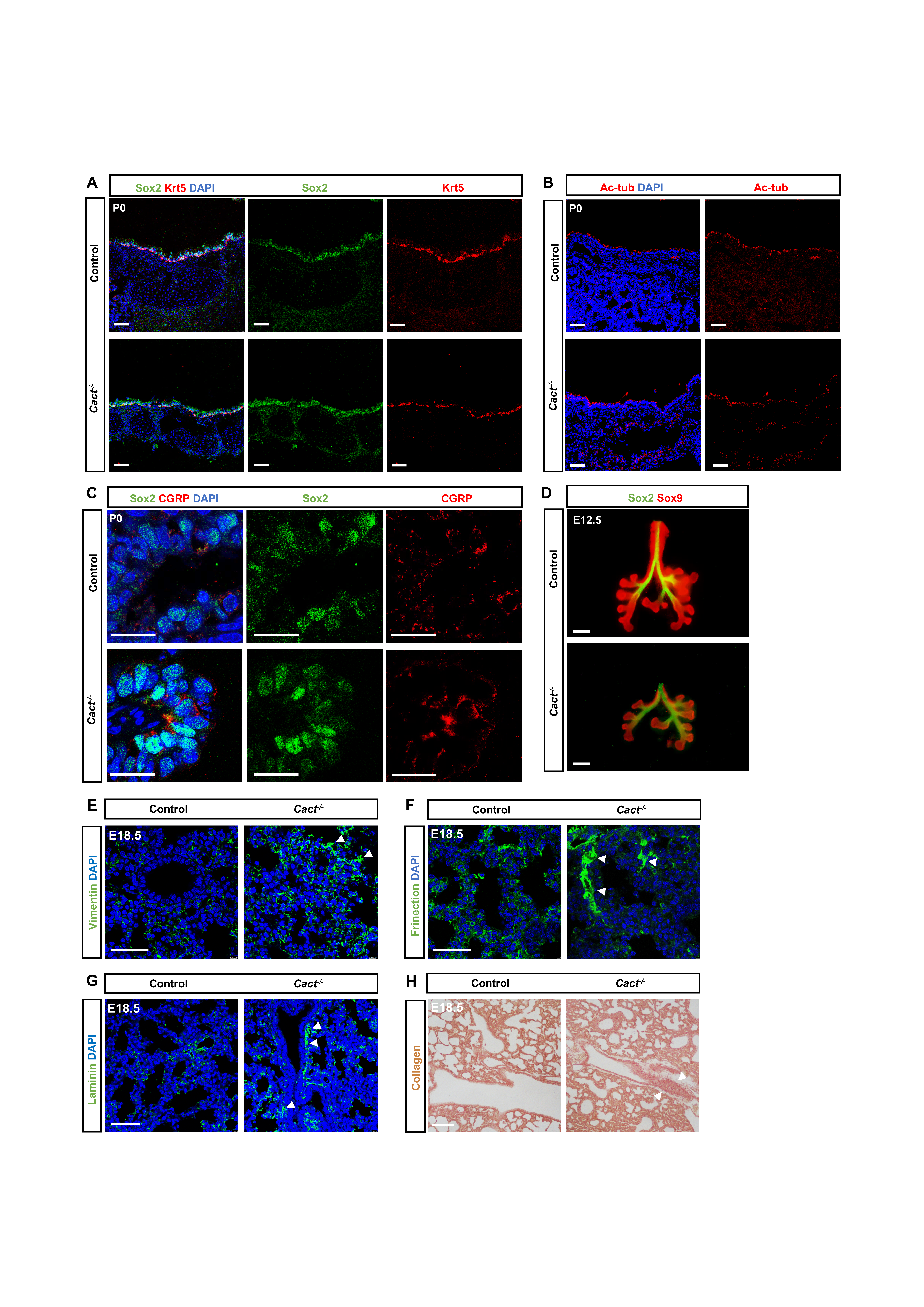

Supplement: Supplementary 1 — Figs. S1 to S8 [file research.0620.f1.zip › Figure S5.TIF]

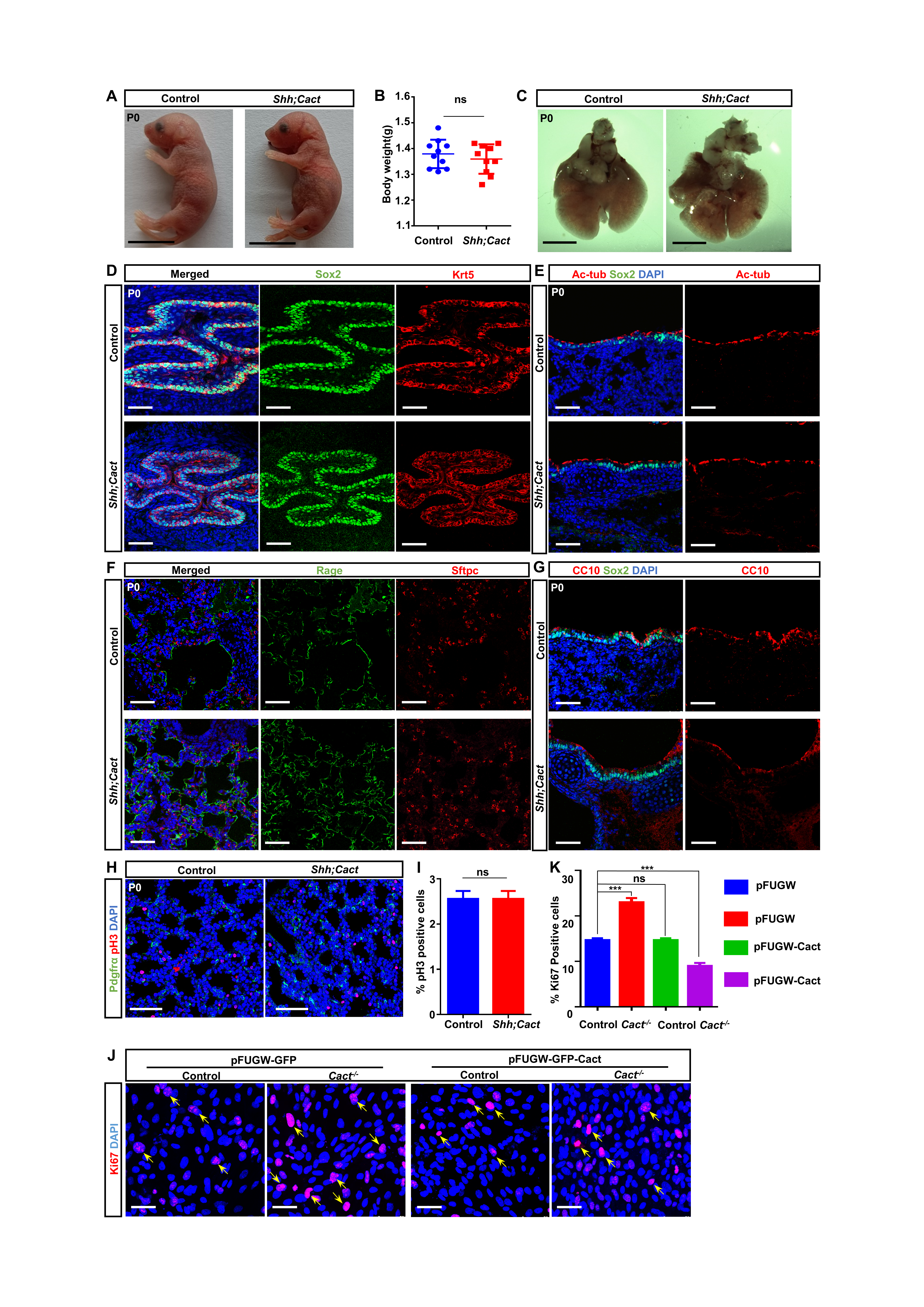

Supplement: Supplementary 1 — Figs. S1 to S8 [file research.0620.f1.zip › Figure S6.TIF]

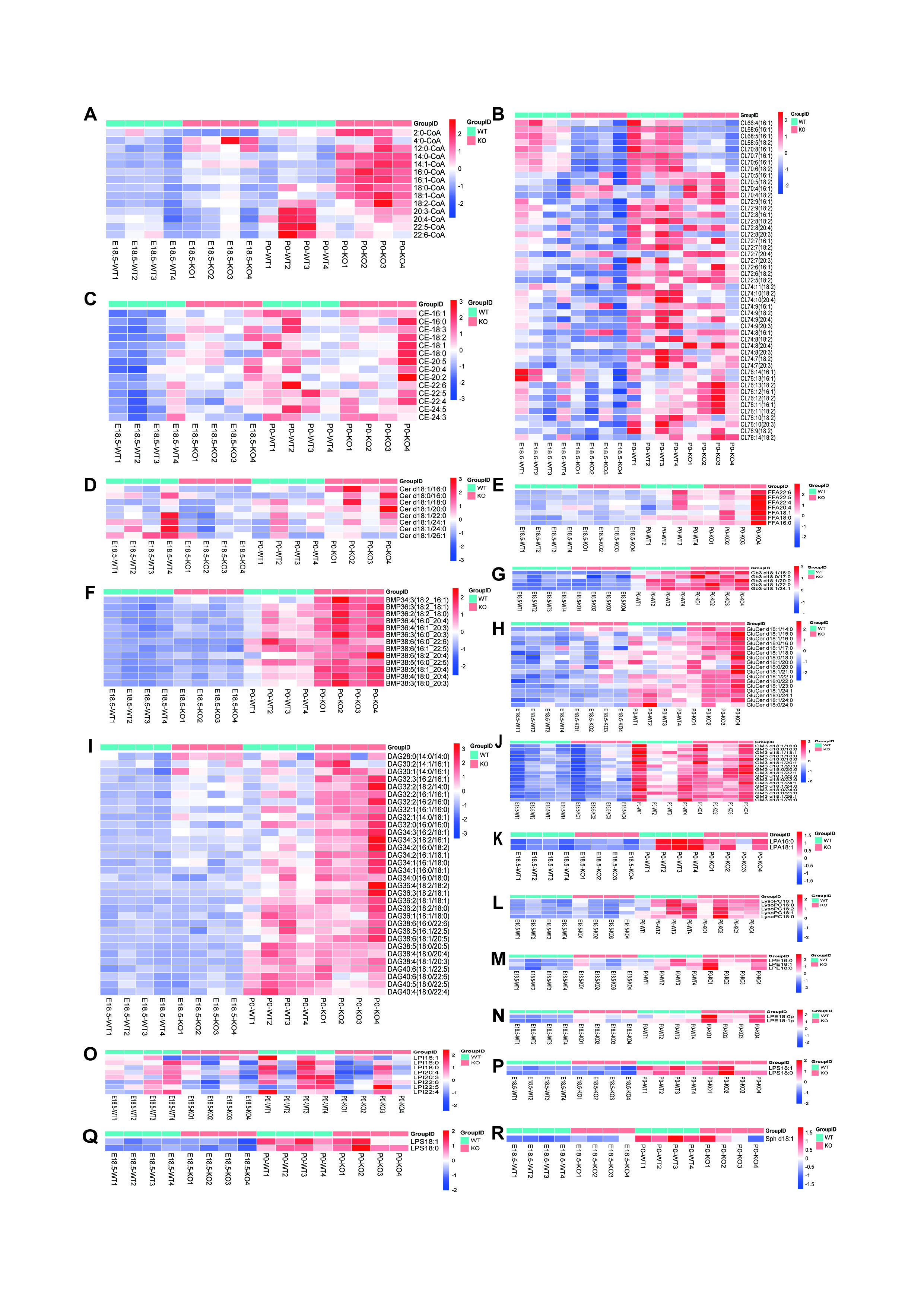

Supplement: Supplementary 1 — Figs. S1 to S8 [file research.0620.f1.zip › Figure S7.tif]

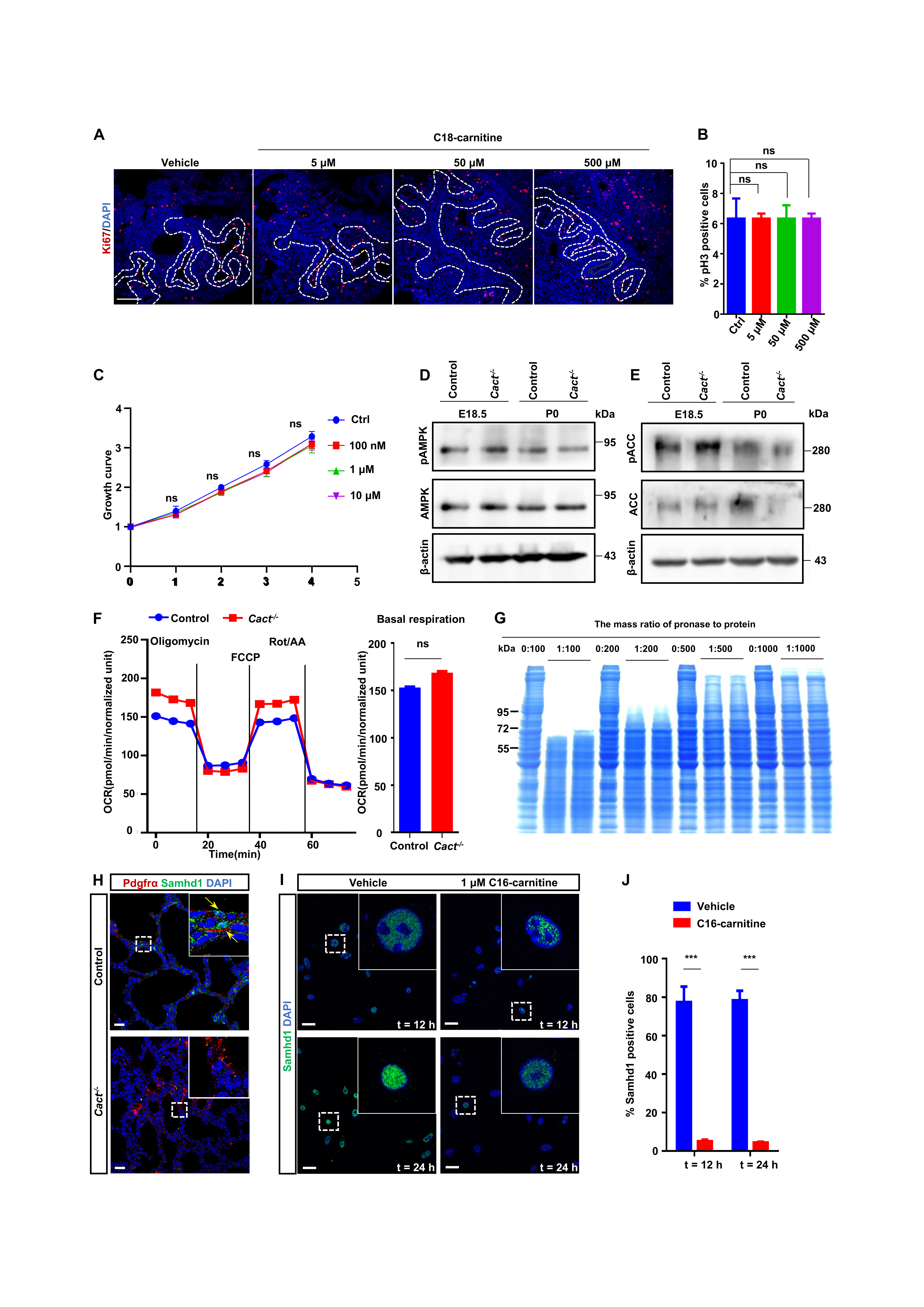

Supplement: Supplementary 1 — Figs. S1 to S8 [file research.0620.f1.zip › Figure S8.TIF]
